# Farnesyl pyrophosphate is a new danger signal inducing acute cell death

**DOI:** 10.1371/journal.pbio.3001134

**Published:** 2021-04-26

**Authors:** Jing Chen, Xiaochen Zhang, Liping Li, Xianqiang Ma, Chunxiao Yang, Zhaodi Liu, Chenyang Li, Maria J. Fernandez-Cabezudo, Basel K. al-Ramadi, Chuan Wu, Weishan Huang, Yong Zhang, Yonghui Zhang, Wanli Liu

**Affiliations:** 1 School of Life Sciences, Institute for Immunology, Ministry of Education Key Laboratory of Protein Sciences, Beijing Advanced Innovation Center for Structural Biology, Collaborative Innovation Center for Diagnosis and Treatment of Infectious Diseases, Beijing Key Lab for Immunological Research on Chronic Diseases, Tsinghua University, Beijing, China; 2 Neuroscience Research Institute and Department of Neurobiology, School of Basic Medical Sciences, Peking University, Key Laboratory for Neuroscience, Ministry of Education/National Health Commission of People’s Republic of China, IDG/McGovern Institute for Brain Research at Peking University, Beijing, China; 3 Tsinghua-Peking Center for Life Sciences, School of Pharmaceutical Sciences, Ministry of Education Key Laboratory of Bioorganic Phosphorus Chemistry & Chemical Biology, Tsinghua University, Beijing Advanced Innovation Center for Human Brain Protection, Beijing, China; 4 School of Medicine, Nankai University, Tianjin, China; 5 Department of Biochemistry and Molecular Biology, College of Medicine and Health Sciences, United Arab Emirates University, Al Ain, United Arab Emirates; 6 Department of Medical Microbiology & Immunology, College of Medicine and Health Sciences, United Arab Emirates University, Al Ain, United Arab Emirates; 7 Zayed Center for Health Sciences, United Arab Emirates University, Al Ain, United Arab Emirates; 8 Experimental Immunology Branch, National Cancer Institute, National Institutes of Health, Bethesda, Maryland, United States of America; 9 Department of Microbiology and Immunology, College of Veterinary Medicine, Cornell University, Ithaca, New York, United States of America; 10 Tsinghua-Peking Center for Life Sciences, Beijing, China; UCSD, UNITED STATES

## Abstract

Cell death is a vital event in life. Infections and injuries cause lytic cell death, which gives rise to danger signals that can further induce cell death, inflammation, and tissue damage. The mevalonate (MVA) pathway is an essential, highly conserved and dynamic metabolic pathway. Here, we discover that farnesyl pyrophosphate (FPP), a metabolic intermediate of the MVA pathway, functions as a newly identified danger signal to trigger acute cell death leading to neuron loss in stroke. Harboring both a hydrophobic 15-carbon isoprenyl chain and a heavily charged pyrophosphate head, FPP leads to acute cell death independent of its downstream metabolic pathways. Mechanistically, extracellular calcium influx and the cation channel transient receptor potential melastatin 2 (TRPM2) exhibit essential roles in FPP-induced cell death. FPP activates TRPM2 opening for ion influx. Furthermore, in terms of a mouse model constructing by middle cerebral artery occlusion (MCAO), FPP accumulates in the brain, which indicates the function of the FPP and TRPM2 danger signal axis in ischemic injury. Overall, our data have revealed a novel function of the MVA pathway intermediate metabolite FPP as a danger signal via transient receptor potential cation channels.

## Abstract 2

Farnesyl pyrophosphate, a metabolic intermediate of the cholesterol synthesis pathway, functions as a novel danger signal to trigger acute cell necrosis, and collaborates with activation of the cation channel TRPM2 to play an important role in brain ischemic injury.

## Introduction

The homeostasis between cell survival and cell death is dynamically regulated throughout the life cycle of an organism [1]. Cell death happens under not only physiological conditions, e.g., development and aging [2], but also pathological conditions including ischemia, inflammation, cancer, neurodegeneration, toxin intake, and many chronic diseases [3,4]. Bioactive mediators released after cell death present “danger signals” to the local microenvironment, which can recruit inflammatory cells and trigger secondary cell death [5–7]. Well-characterized danger signal molecules include the non-histone DNA-binding nuclear protein high mobility group protein B1 (HMGB1) and the cellular energy supplier ATP, both of which can trigger inflammation. HMGB1 promotes dendritic cell maturation and antigen presentation by activating Toll-like receptors [8,9], while ATP activates the NLRP3 inflammasome via purinergic receptors to trigger the release of the pro-inflammatory cytokine IL1β [6,10,11]. Specifically, extracellular ATP activates the P2X7 receptor in antigen-presenting cells, leading to plasma membrane depolarization and Ca^2+^ influx, then pannexin-1 mediates the formation of pores permeable to large molecules, followed by cell lysis [12–14].

Isoprenoid biosynthesis is universal and highly conserved across all living organisms, and isoprenoids are one of the largest groups of biomolecules with a wide range of biological functions [15]. In mammals, the mevalonate (MVA) pathway is utilized to synthesize isoprenoids from Acetyl-CoA to provide farnesyl pyrophosphate (FPP), geranyl-geranyl pyrophosphate (GGPP), ubiquinone, and cholesterol, mediating protein prenylation, functioning in electron transport chain, and as membrane component, respectively [16,17]. In detail, the MVA pathway first produces the building blocks isopentenyl pyrophosphate (IPP) and dimethylallyl pyrophosphate (DMAPP). Then, the 5-carbon chain from IPP is added to geranyl pyrophosphate (GPP) and FPP in the cytosol and mitochondria, which yields 10-, 15-, and 20-carbon-long isoprenoids. Sharing Acetyl-CoA produced by glycolysis with tricarboxylic acid cycle and fatty acid synthesis, the MVA pathway is dynamically regulated based on cell status. Indeed, efficient up-regulation of the MVA pathway activity has been reported in tumor cells via a Myc-driven manner, in activated T cells in an Akt-mTOR-SREBP-dependent way [18] and in hypoxic cells in a HIF-1-dependent process [19]. Consistently, FPP and GGPP accumulation can be readily detected in male patients with Alzheimer disease (AD) and those with hyperglycemia accompanied by chronic inflammation [20,21]. All shreds of evidence drive our curiosity about the downstream effects of excessive isoprenoids and the underlying mechanisms of ligand sensing and signaling activation.

Here, we report that FPP, containing a lipophilic hydrocarbon chain and lipophobic pyrophosphate head, functions as a novel danger signal that induces acute cell death through melastatin-related transient receptor potential cation channels (TRPMs). In brief, we synthesized all 5 metabolic intermediates of the MVA pathway and found that only FPP and GGPP led to acute cell death in a dose-dependent manner. This cytotoxic effect was highly specific and dependent on the structure of FPP, especially the length of the unsaturated hydrocarbon chain and the bulky structure of the pyrophosphate group. FPP-induced cytotoxicity is independent of its known downstream metabolites and distinct from the conventional cell death but relies on extracellular calcium influx to the cytosol. By knocking out the frequently used calcium channels in mammalian cells including the Orai, P2RX, and the TRP family channels, we found that transient receptor potential melastatin 2 (TRPM2) was involved in FPP-induced cell death. Indeed, electrophysiological recordings showed that FPP evoked TRPM2 channel opening. Moreover, triphenylphosphine oxide, Simvastatin, and Zoledronic acid treatment alleviated cerebral infarction in a mouse model of middle cerebral artery occlusion (MCAO). Overall, our data identify FPP as a newly identified danger signal to trigger acute cell death and suggest that the FPP/TRPM2 signaling axis and the MVA pathway exhibit important roles in brain ischemia and potentially neurodegenerative diseases.

## Results

### FPP induces acute cell death

**Fig 1:**
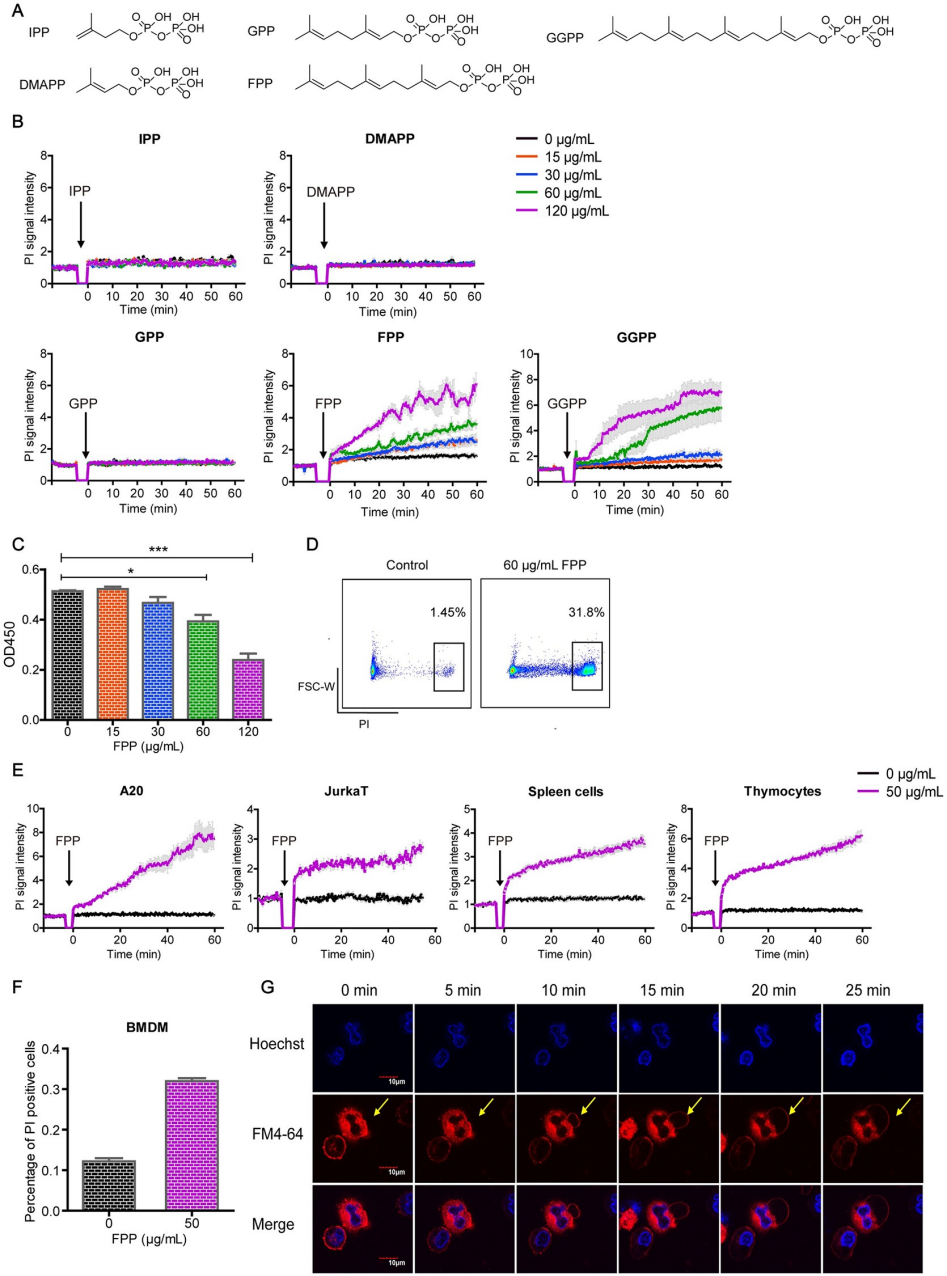
FPP in MVA pathway induces acute cell death. (A) Molecular structures of intermediate metabolites in MVA pathway IPP, DMAPP, GPP, FPP, and GGPP. (B) PI signal increase under fluorescent microplate reader after IPP, DMAPP, GPP, FPP, and GGPP treatment. Black arrows indicate the time points at which different metabolites were added. Each treatment has 3 to 4 replicates. Bars denote mean ± SEM (SEM in gray). There are no significant differences between 0 μg/mL, 15 μg/mL, and 30 μg/mL FPP treatments. Significant difference in 60 μg/mL FPP group compared to the 0 μg/mL FPP group shows up from 25 minutes on (*p* < 0.05). Significant difference in 120 μg/mL FPP group compared to the 0 μg/mL FPP group shows up from 10 minutes on (*p* < 0.01). There are no significant differences between 0 μg/mL, 15 μg/mL, and 30 μg/mL GGPP treatments. Significant difference in 60 μg/mL GGPP group compared to the 0 μg/mL GGPP group shows up from 30 minutes on (*p* < 0.05). Significant difference in 120 μg/mL GGPP group compared to the 0 μg/mL GGPP group shows up from 15 minutes on (*p* < 0.01). All the others show no significant differences. (*p* > 0.05). Two-way ANOVA analysis is used. (C) CCK8 signal change after FPP treatment. Bars denote mean ± SEM. Unpaired 2-tailed Student *t* test were used. (D) PI-positive cell percentage was measured by flow cytometry with and without FPP treatment. (E) PI signal increases under a fluorescent microplate reader after B cell line A20 cells, Jurkat T cell line, primary spleen cells, and primary thymocytes were treated with FPP. Black arrows mean the time points at which FPP was added. Bars denote mean ± SEM. Significant difference between FPP treatment and the control group shows up at time 10 minutes in A20 cells (*p* < 0.05). Significant difference between FPP treatment and the control group shows up at time 0 minute in Jurkat T cells, spleen cells, and thymocytes (*p* < 0.001). Two-way ANOVA analysis is used. (F) BMDMs were stained with Hoechst and treated with FPP in the presence of PI. One hour later, cells were imaged and PI-positive cell number versus Hoechst cell number was defined as percentage of PI-positive cells (*p* < 0.01). (G) P815 cell images under Olympus confocal microscopy after incubation with 60 μg/mL FPP. Cells were stained with Hoechst (in blue) and FM4-64 (in red). Scale bars are 10 μm. Data are representative of at least 2 independent experiments in (B) to (E) and (G). The original data in this figure except Fig 1D can be found in S1 Data. The original data in Fig 1D can be found in S3 Data. BMDM, bone marrow–derived macrophage; CCK8, Cell Counting Kit-8; DMAPP, dimethylallyl pyrophosphate; FPP, farnesyl pyrophosphate; GGPP, geranyl-geranyl pyrophosphate; GPP, geranyl pyrophosphate; IPP, isopentenyl pyrophosphate; PI, propidium iodide.

Our initial aim was to determine whether any of the 5 MVA pathway metabolic intermediates can regulate the activation and function of immune cells. To this end, IPP, DMAPP, GPP, FPP, and GGPP were chemically synthesized (Fig 1A, S1 Text) and incubated in vitro with the mouse mastocytoma cell line, P815. When using propidium iodide (PI) staining to gate on live cells for downstream functional analyses, we unexpectedly found that both FPP and GGPP treatments resulted in an instant and significant cell death (indicated by PI signal increase) in a dose-dependent manner, while DMAPP, IPP, and GPP treatments did not show such effect (Fig 1B). The chemical synthesis of GGPP is more difficult than FPP, and the cell death phenotype triggered by FPP was more stable than that by GGPP, which compelled us to focus on FPP in subsequent experiments. The cytotoxic effect of FPP was further confirmed by Cell Counting Kit-8 (CCK8)-based cell viability assay (Fig 1C) and PI-based flow cytometric analysis (Fig 1D). FPP-induced cell death was also observed in A20 B cell line, Jurkat T cell line, primary spleen cells, primary thymocytes, and bone marrow–derived macrophages (BMDMs) (Fig 1E and 1F). To understand the cellular effects of FPP in more detail, we stained the nuclear and plasma membranes to capture their morphological changes after FPP treatment by confocal microscopy. Similar to the typical morphological changes during necrosis [22], within 5 minutes after FPP addition, P815 cells initiated cytoplasmic swelling, which increased over time, until finally the plasma membrane was ruptured (Fig 1G). Thus, the MVA pathway metabolic intermediate FPP is a danger signal that can trigger acute cell death manifesting cell swelling and loss of plasma membrane integrity.

### Structural dependence of FPP-induced acute cell death

**Fig 2:**
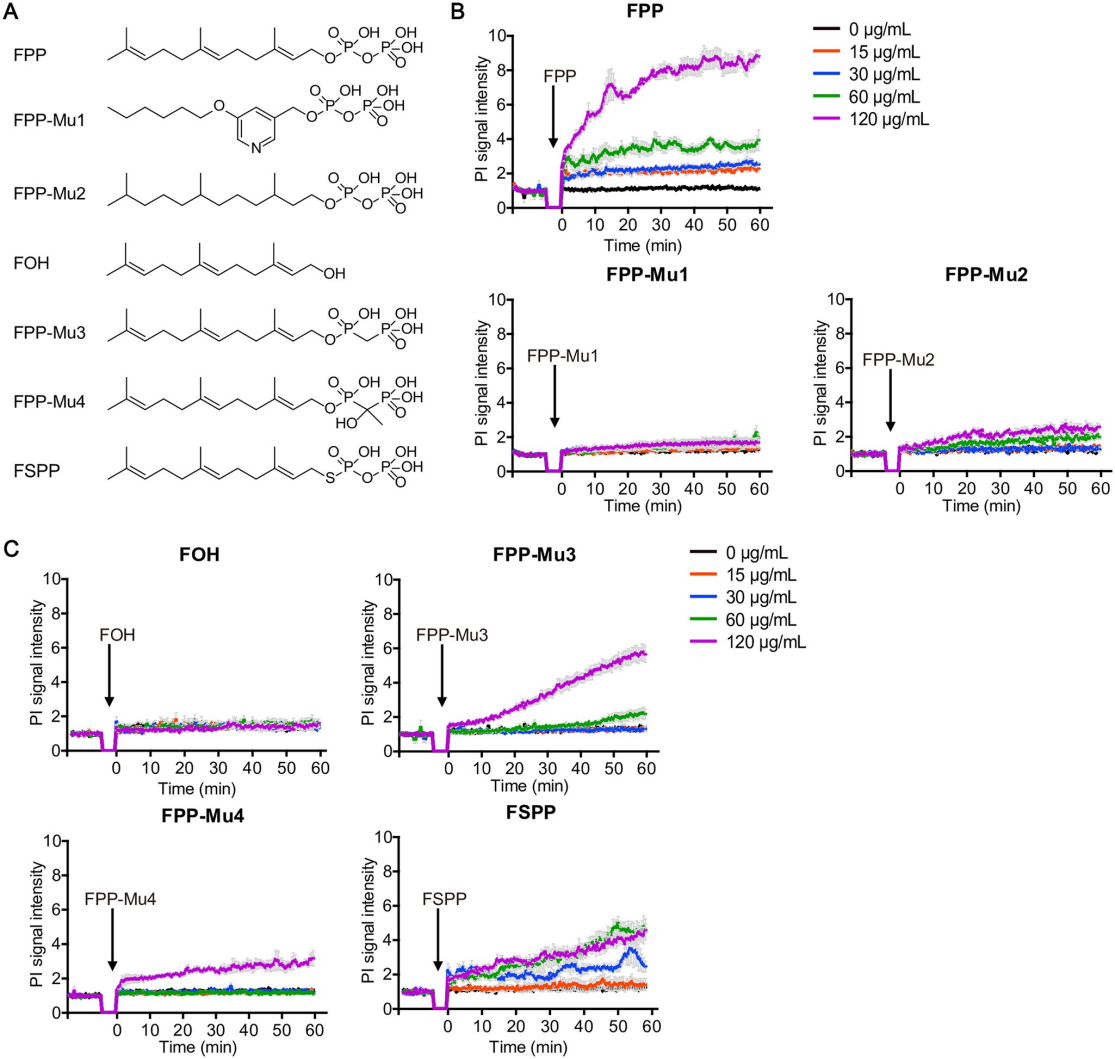
Both the length of the hydrocarbon chain and the pyrophosphate group are crucial for FPP-induced acute cell death. (A) Molecular structures of FPP and different FPP analogs including FPP analog 1/2/3/4, FOH, and FSPP. (B, C) Cytotoxic effects of different FPP analogs measured by PI signal increase under fluorescent microplate reader. Black arrows indicate the time points at which different metabolites were added. Each treatment had 3 to 4 replicates. Bars denote mean ± SEM (SEM in gray). Data are representative of at least 2 independent experiments in (B) and (C). There are no significant differences between 0 μg/mL and 15 μg/mL FPP treatments. Significant difference in 30 μg/mL FPP group compared to the 0 μg/mL FPP group shows up from 47 minutes on (*p* < 0.05). Significant difference in 60 μg/mL FPP group compared to the 0 μg/mL FPP group shows up from 1 minute on (*p* < 0.01). Significant difference in 120 μg/mL FPP group compared to the 0 μg/mL FPP group shows up from 1 minute on (*p* < 0.001). Significant difference between 60 μg/mL FPP-Mu2 and the control shows up from 39 minutes on (*p* < 0.05). Significant difference between 120 μg/mL FPP-Mu2 and the control shows up from 10 minutes on (*p* < 0.01). Significant difference between 60 μg/mL FPP-Mu3 and the control shows up from 54 minutes on (*p* < 0.05). Significant difference between 120 μg/mL FPP-Mu3 and the control shows up from 12 minutes on (*p* < 0.05). Significant difference between 120 μg/mL FPP-Mu4 and the control shows up from 2.5 minutes on (*p* < 0.01). Significant difference between 30 μg/mL FSPP and the control shows up from 43 minutes on (*p* < 0.05). Significant difference between 60 μg/mL FSPP and the control shows up from 16 minutes on (*p* < 0.05). Significant difference between 120 μg/mL FSPP and the control shows up from 10 minutes on (*p* < 0.05). All the others show no significant difference (*p* > 0.05). Two-way ANOVA analysis is used. All experiments are done with P815 cells. All the original data can be found in S1 Data. FOH, farnesol; FPP, farnesyl pyrophosphate; FSPP, farnesyl S-thiolodiphosphate; PI, propidium iodide.

We speculated that the length of the unsaturated hydrocarbon chain might be important for the cytotoxic effect, given that only FPP and GGPP, but not DMAPP, IPP, or GPP, triggered acute cell death. To investigate the structural requirements of FPP-induced cell death, a series of FPP analogs were synthesized (Fig 2A, S1 Text). FPP analog 1 (FPP-Mu1), carrying a pyridine ring instead of the linear unsaturated hydrocarbon chain, totally abolished the cytotoxic effect (Fig 2B). FPP analog 2 (FPP-Mu2), retaining the same hydrocarbon chain length but carrying saturated hydrocarbon bonds instead of the original unsaturated hydrocarbon bonds, failed to induce cytolysis as well (Fig 2B). The 2 analogs together demonstrated the key roles of the length and structure of the unsaturated hydrocarbon chain in the cytotoxic effect of FPP. When the pyrophosphate head was substituted with a hydroxyl group (−OH), the resultant compound farnesol (FOH) also totally lost the capability to induce cell death, suggesting that the cytotoxic effect of FPP might be dependent on the presence of an intact pyrophosphate bulky head structure (Fig 2C). Indeed, FPP analog 3 (FPP-Mu3), in which the oxygen between the 2 phosphate groups was changed to carbon, and FPP analog 4 (FPP-Mu4), in which hydroxyl and methyl groups were further introduced to substitute the hydrogen equivalents for the central carbon atom (Fig 2A), retained cytotoxic effects, despite significantly weaker activities as compared to FPP (Fig 2C). As a further validation, even when we changed the oxygen atom linking the head and tail to sulfur resulting in farnesyl S-thiolodiphosphate (FSPP), we only observed a certain level cytotoxic effect at intermediate and high concentrations (Fig 2C). These data suggest that the cytotoxic effect of FPP is dependent on the integrity of both the hydrophobic polyunsaturated hydrocarbon chain and the charged and hydrophilic pyrophosphate bulky head.

### The downstream metabolites and conventional cell death pathways are not required for FPP-induced cell death

**Fig 3:**
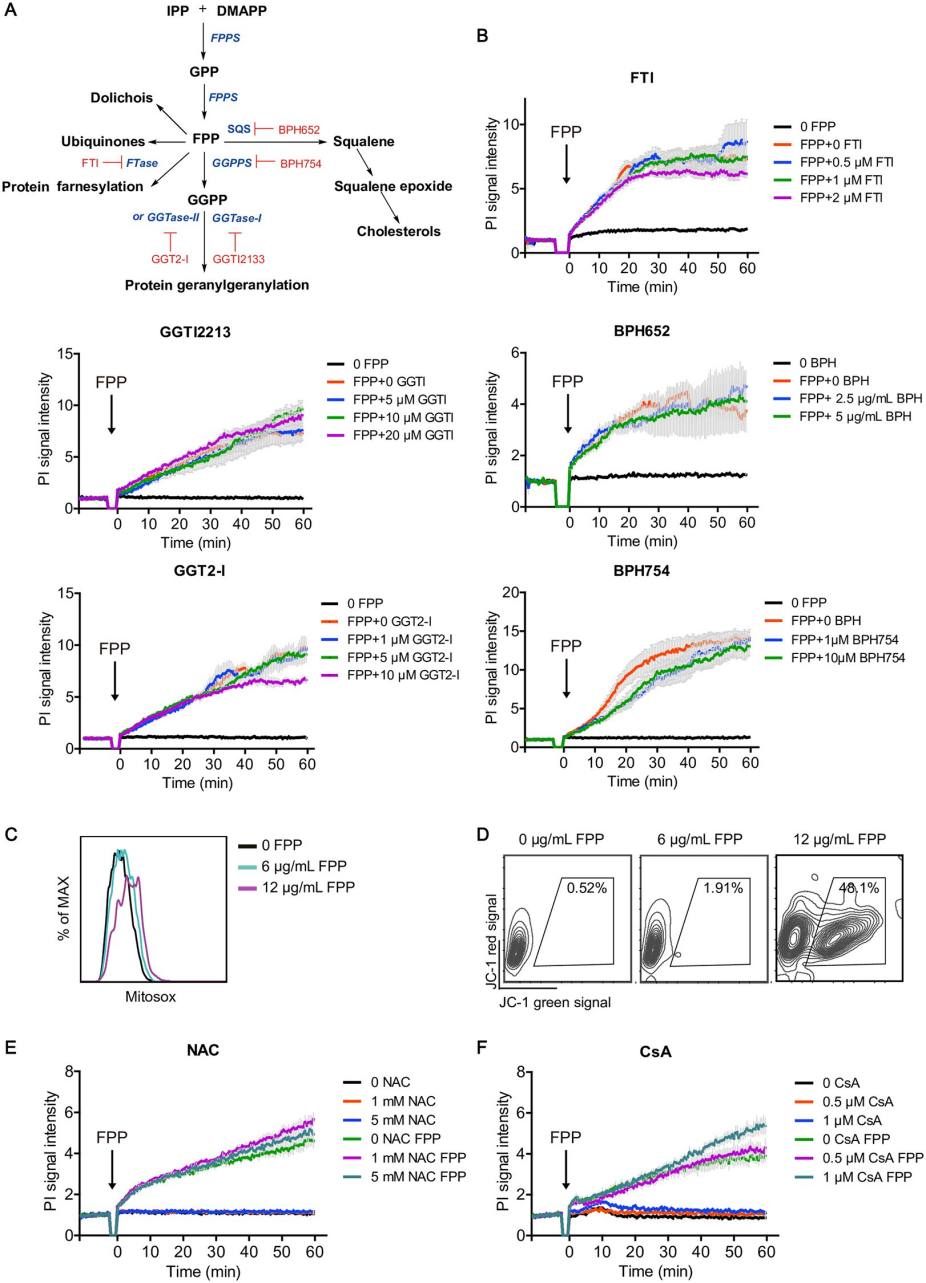
FPP downstream metabolic pathways and conventional cell death pathways are not required in FPP-induced cell death. (A) Schematic view of FPP upstream and downstream metabolic pathway and their inhibitors. Enzymes are in blue, and inhibitors are in red. (B) Effects of FPP downstream pathway inhibitors on FPP-induced cell death. Black arrows indicate the time points at which different reagents were added. Bars denote mean ± SEM (SEM in gray). (C) Intracellular ROS indicated with mitosox stain after FPP treatment. (D) Mitochondrial potential decrease indicated with jc-1 stain after FPP treatment. (E, F) Effects of ROS scavenger NAC (E) and mitochondrial permeability inhibitor CsA (F) on FPP-induced cell death. Each treatment has 3 to 4 replicates. Bars denote mean ± SEM (SEM in gray). Data are representative of at least 2 independent experiments in (B) to (E). No significant differences between the inhibitor treatment groups and the no inhibitor treatment group have been observed in (B), (E), and (F) (*p* > 0.05). Two-way ANOVA analysis is used. All experiments are done with P815 cells. Original data in Fig 3B, 3E, and 3F can be found in S1 Data. Original data in Fig 3C and 3D can be found in S3 Data. CsA, cyclosporin A; DMAPP, dimethylallyl pyrophosphate; FPP, farnesyl pyrophosphate; FPPS, farnesyl diphosphate synthase; FTI, farnesyl transferase inhibitor; GGPP, geranyl-geranyl pyrophosphate; GPP, geranyl pyrophosphate; IPP, isopentenyl pyrophosphate; NAC, N-acetyl-l-cysteine; ROS, reactive oxygen species.

To explore the molecular mechanisms underlying FPP-induced cell death, we first examined different steps of the downstream MVA pathway by blocking squalene synthesis with BPH652, farnesyl decoration with farnesyl transferase inhibitor (FTI), GGPP synthase with BPH754, or geranylgeranyl decoration with GGT2I and GGTI2133, respectively (Fig 3A). However, none of these inhibitors affected FPP-induced cell death (Fig 3B). Mitochondria play an important role in both apoptosis and programmed necrosis [23]. FPP treatment triggered mitochondrial reactive oxygen species (ROS) production and membrane potential loss (Fig 3C and 3D). To determine whether mitochondria function downstream of FPP, we use ROS scavenger N-acetyl-l-cysteine (NAC) and the mitochondrial pore blocker cyclosporin A (CsA) to block mitochondrial activities, but found them with no effects on FPP-induced cell death (Fig 3E and 3F). Consistently, neither Bax/Bak knockout nor Bcl2 overexpression affected FPP-induced cell death (S1A–S1D Fig). We also tested other inhibitors targeting the necroptosis pathway, small GTPases, and caspases [24], and again, no effects on FPP-induced cell death were detected (S2A–S2C Fig). These data suggest that neither MVA pathway–related signaling (e.g., cholesterol and prenylated small GTPases) nor conventional cell death pathway may play a significant role in mediating the cytotoxic effect of FPP.

### Extracellular calcium is vital for FPP-induced acute cell death

**Fig 4:**
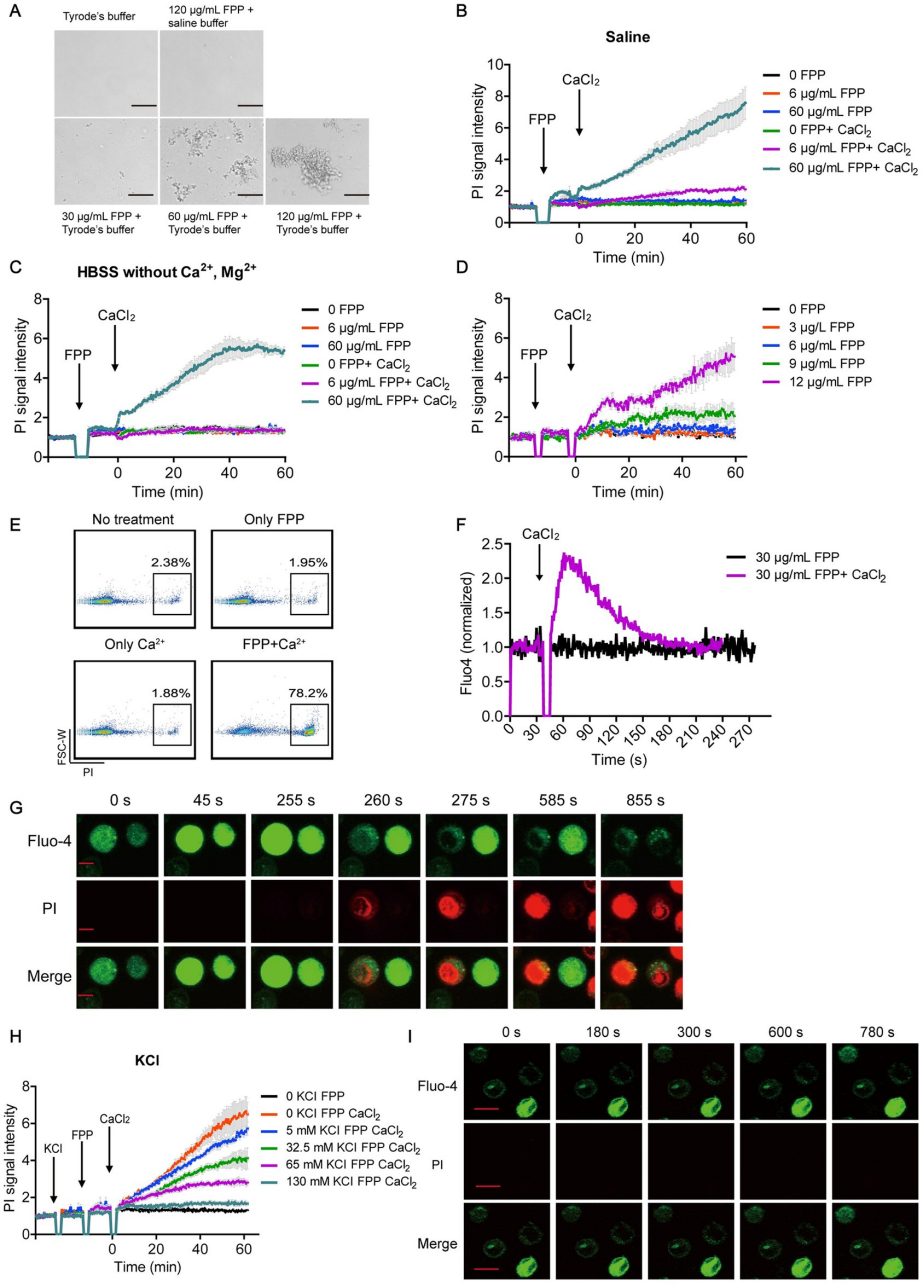
Extracellular calcium influx is vital for FPP-induced cell death. (A) Precipitates observed when different concentrations of FPP in H_2_O were added to Tyrode’s buffer or saline buffer. The scale bar indicates 100 μm. (B, C) Cells in saline buffer (B) or HBSS without calcium and magnesium (C) were treated with FPP, and PI signal changes in the presence or absence of calcium were measured under fluorescent microplate reader. Black arrows indicate the time points at which FPP is added. Bars denote mean ± SEM (SEM in gray). Significant difference between 60 μg/mL FPP with CaCl_2_ and the no CaCl_2_ control in saline buffer shows up after 15 minutes (*p* < 0.05). Significant difference between 60 μg/mL FPP with CaCl_2_ and the no CaCl_2_ control in HBSS buffer shows up after 15 minutes (*p* < 0.01). All the others show no significant differences (*p* > 0.05). Two-way ANOVA analysis is used. (D) PI signal change under fluorescent microplate reader in saline buffer after being treated with different concentrations of FPP. Significant difference between 9 μg/mL FPP and the control group shows up from 48 minutes on (*p* < 0.05). Significant difference between 12 μg/mL FPP and the control group shows up from 19 minutes on (*p* < 0.01). All the others show no significant differences (*p* > 0.05). Two-way ANOVA analysis is used. (E) PI-positive cell percentage measured under flow cytometry after cells were treated with FPP in the presence or absence of calcium. (F) Flow cytometry measurements of intracellular calcium level measured by fluo4 signal change before and after FPP treatment. (G) Calcium entry and cell death status monitored after FPP treatment under confocal microscopy. Green color indicates fluo4 staining, and red color indicates PI staining. (H) PI signal change in the presence of different concentration of KCl after FPP treatment. Black arrows indicate the time points at which different reagents were added. Bars denote mean ± SEM (SEM in gray). Significant difference between 0 KCl FPP and 0 KCl FPP CaCl_2_ group shows up from 9 minutes on (*p* < 0.05). Significant difference between 0 KCl FPP and 5 mM KCl FPP CaCl_2_ group shows up from 10.5 minutes on (*p* < 0.05). Significant difference between 0 KCl FPP and 32.5 mM KCl FPP CaCl_2_ group shows up from 19 minutes on (*p* < 0.05). Significant difference between 0 KCl FPP and 65 mM KCl FPP CaCl_2_ group shows up from 19 minutes on (*p* < 0.05). All the others show no significant differences (*p* > 0.05). Two-way ANOVA analysis is used. (I) Calcium entry and cell death status after FPP treatment under confocal microscopy in the presence of KCl. Green color indicates fluo4 staining, and red color indicates PI staining. Data are representative of at least 2 independent experiments in (B) to (I). All experiments are done with P815 cells. Original data in Fig 4 B–D and 4H can be found in S1 Data. Original data in Fig 4E can be found in S3 Data. Original data in Fig 4F can be found in S4 Data. FPP, farnesyl pyrophosphate; PI, propidium iodide.

Of note, Tyrode’s buffer (137 mM NaCl, 2.7 mM KCl, 1.0 mM MgCl_2_, 1.8 mM CaCl_2_, 20 mM HEPES, and 5.6 mM glucose, pH 7.4) used in the aforementioned cytotoxicity assays contained calcium at the physiological concentration and manifested precipitation in the presence of high concentrations of FPP (Fig 4A). This prompted us to hypothesize that preexisting calcium-induced FPP precipitation may affect the efficiency of FPP-induced cell death. Therefore, we switch to buffer without calcium, namely saline buffer or HBSS without calcium or magnesium, to perform the same experiment. Unexpectedly, cells in these calcium-free buffers showed no PI signal increase, even in the presence of high concentrations of FPP (Fig 4B and 4C), while supplementing these calcium-free buffers with 2 mM CaCl_2_ fully restored the cytotoxic activity of FPP (even at a low concentration of 9 μg/mL) (Fig 4B–4D). Thus, extracellular calcium plays a vital role in FPP-induced cell death, which was further confirmed by PI-positive cell counting under the same conditions by flow cytometry (Fig 4E). Notably, not only CaCl_2_ but also SrCl_2_ and BaCl_2_ promoted acute cell death after FPP treatment, albeit with different potencies and optimal working concentrations. MgCl_2_ was, however, unable to promote FPP-induced cell death, even though magnesium is in the same periodic table group as calcium, strontium, and barium (S3A and S3B Fig).

It is possible that FPP can regulate the transportation of extracellular calcium into cells. Indeed, we observed a stiff calcium influx peak in flow cytometry immediately following the addition of extracellular CaCl_2_ to FPP-pretreated cells (Fig 4F). Moreover, this FPP-induced immediate calcium influx occurs prior to cell death as indicated by PI permeabilization (Fig 4G), suggesting that the influxed calcium functions downstream of FPP and mediates FPP-induced cell death.

Cytosolic calcium overload can induce cell death upon ATP treatment [14,25], and it has been reported that the presence of extracellular potassium can inhibit ATP-triggered pyroptosis [26,27]. Similarly, in our system, extracellular potassium could inhibit FPP-induced cell death in a dose-dependent manner (Fig 4H), in which 135 mM KCl blocked calcium entry and the subsequent cell death in P815 cells (Fig 4I). To further explore the mechanism of FPP-induced calcium influx, we first examined the role of the endoplasmic reticulum (ER)-mediated intracellular calcium signaling. Using 2-APB, an inhibitor known to block ER calcium efflux into the cytoplasmic compartment, we found that blocking STORE-operated calcium signaling does not affect FPP-induced cell death (S3C Fig), although it significantly reduced calcium influx in DT40 B cells following antigen stimulation (S3D Fig). Furthermore, knocking out stromal-interacting molecule 1 (STIM1) or inositol trisphosphate receptor (IP3R), proteins that are critical in ER-mediated calcium signaling upon B cell receptor (BCR) stimulation [28], does not affect FPP-induced cell death in DT40 B cells (S3E Fig). These data suggest that FPP-induced cell death is independent of ER-mediated intracellular calcium signaling. Therefore, FPP-induced cell death mainly relies on extracellular calcium influx, but not ER-mediated intracellular calcium efflux.

### FPP induces cell death through the activation of TRPM2

To determine which ion channels are responsible for FPP-induced calcium influx, we knocked out the well-characterized calcium channels orai1/2/3 individually, but found no attenuating effects of their absence on FPP-induced cell death (S4A Fig). Neither did the absence of the 2 pore potassium channels, TREK1 and TRAAK, showed any effects (S4B Fig), even though these 2 channels can be opened by polyunsaturated fatty acids such as arachidonic acid [29]. P2X channels, used by a well-characterized danger signal, ATP, to induce cell death, were also not involved in cell death triggered by FPP (S4C and S4D Fig).

**Fig 5:**
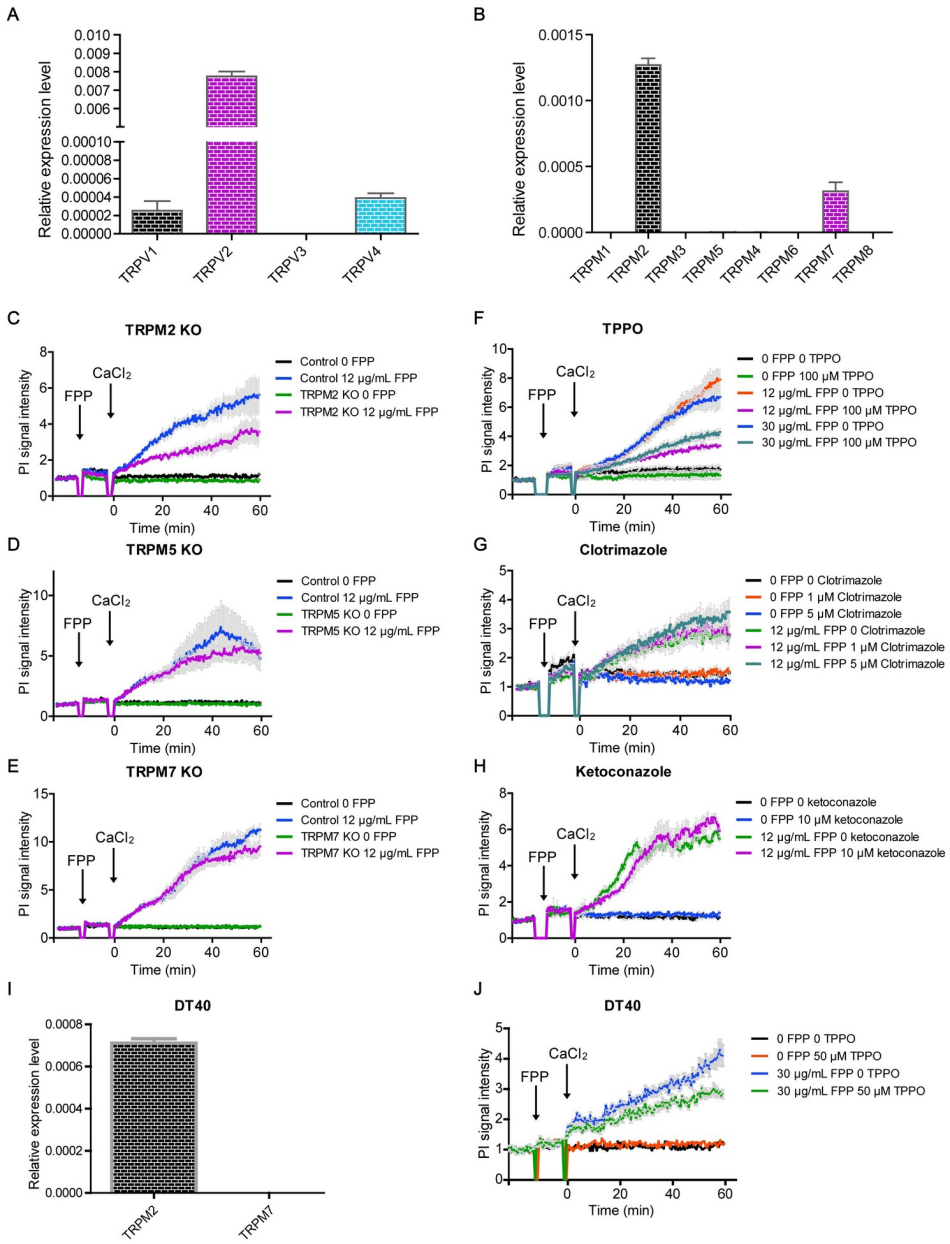
FPP-induced cell death is dependent on TRPM2 channel. (A) Relative mRNA level of TRPV1/2/3/4 in mast cell detected by qPCR. (B) Relative mRNA level of TRPM1/2/3/4/5/6/7/8 in mast cell detected by qPCR. (C–E) TRPM2 (C), TRPM5 (D), and TRPM7 (E) KO and their effect on FPP-induced cell death. Each treatment had 3 to 4 replicates. Bars denote mean ± SEM (SEM in gray). The first black arrow indicates the addition of FPP, and the second black arrow indicates the addition of CaCl_2_. Significant difference between control FPP and TRPM2 KO FPP group shows up from 40 minutes on (*p* < 0.05). Two-way ANOVA analysis is used. (F–H) TRPM inhibitors TPPO (F), clotrimazole (G), and ketoconazole (H) were used to disturb FPP-induced cell death. Each treatment had 3 to 4 replicates. Bars denote mean ± SEM (SEM in gray). The first black arrow indicates the addition of FPP, and the second black arrow indicates the addition of CaCl_2_. Significant difference between 12 μg/mL FPP 0 TPPO and 12 μg/mL FPP 100 μM TPPO group shows up from 30 minutes on (*p* < 0.05). Significant difference between 30 μg/mL FPP 0 TPPO and 30 μg/mL FPP 100 μM TPPO group shows up from 28.5 minutes on (*p* < 0.05). Fig 5 A–H is done with P815 cells. (I) TRPM2 and TRPM7 expression level in DT40 cells were checked with qPCR. (J) TPPO inhibit FPP-induced DT40 cell death. DT40 cells were preincubated with 50 μM TPPO for 45 minutes and then treated with 30 μg/mL FPP and CaCl_2_. PI signal increase under fluorescent microplate reader. Black arrows indicate the time points at which different metabolites were added. Each treatment had 3 to 4 replicates. Bars denote mean ± SEM (SEM in gray). Significant difference in 50 μM TPPO FPP group compared to the 0 TPPO FPP group shows up from 30 minutes on (*p* < 0.05). Two-way ANOVA analysis is used. Data are representative of at least 2 independent experiments in (B) to (J). All the original data can be found in S1 Data. FPP, farnesyl pyrophosphate; KO, knockout; PI, propidium iodide; qPCR, quantitative PCR; TRPM2, transient receptor potential melastatin 2.

To further explore the underlying mechanism of FPP-induced calcium influx, we focused on another important group of ion channels, transient receptor potential (TRP) channels, which are potent contributors to extracellular calcium entry into the cytoplasmic compartment [30,31]. FPP has been reported to activate TRPV3, but not TRPA1, TRPV1, TRPV2, TRPV4, or TRPM8 [32]. In P815 mast cells used for the aforementioned FPP-induced cell death assays, we found minimal TRPV3 but high levels of TRPV2 expression (Fig 5A). Since FPP activates TRPV3 but not TRPV2, it is unlikely that FPP induced cell death through the TRPV channels in P815 cells. When probing for TRPM channels in P815 cells, we detected high TPRM2, low TRPM7, and minimal TRPM5 expression (Fig 5B). To determine whether FPP signals through these TRPM channels to induce cell death, we generated P815 cells deficient in TRPM2, TRPM5, and TRPM7, respectively, and found that FPP failed to effectively activate cell death only in the absence of TRPM2 (Fig 5C–5E). TRPM2 is a nonselective calcium permeable cation channel that is involved in chronic inflammation, neurodegeneration, and oxidative stress [33]. Chemical antagonists for TRPM channels have been developed, and we tested the effects of 3 known TRPM inhibitors including TPPO, clotrimazole, and ketoconazole. We found that TPPO inhibited FPP-induced cell death (Fig 5F–5J).

**Fig 6:**
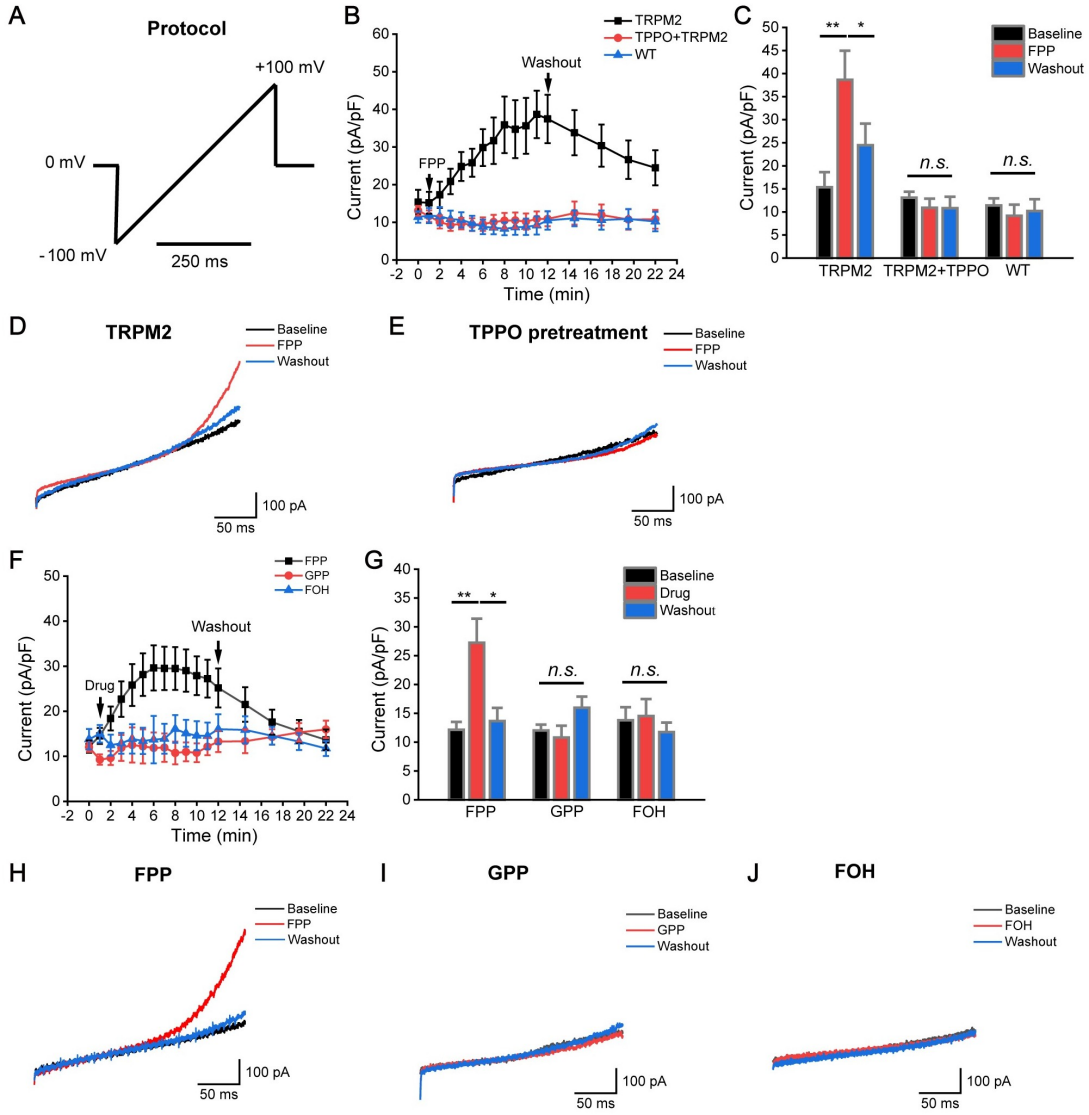
FPP evokes TRPM2 current. (A) A voltage ramp protocol lasting 250 ms from −100 mV to +100 mV. (B) Current change of TRPM2 channels after FPP addition or washout as indicated with black arrows. (C) Current density of TRPM2 overexpressed HEK293 cell before FPP treatment, during FPP application (at 11 minutes) and washout (at 22 minutes). Bars denote mean ± SEM. (*n* = 7 for TRPM2, *n* = 9 for TPPO+TRPM2, and *n* = 13 for WT). (D) The representative TRPM2 current trace elicited by the voltage ramp after FPP stimulation. (E) The representative TRPM2 current trace elicited by the voltage ramp after FPP stimulation in the presence of TPPO pretreatment. (F) Current change of TRPM2 channels after FPP, GPP, and FOH addition or washout as indicated with black arrows. (G) Current density of TRPM2 overexpressed HEK293 cell before FPP, GPP, or FOH treatment, during the reagent application (at 11 minutes) and washout (at 22 minutes). Bars denote mean ± SEM. (*n* = 8 for FPP, *n* = 11 for GPP, *n* = 10 for FOH). (H–J) The representative TRPM2 current trace elicited by the voltage ramp after FPP (H), GPP (I), or FOH (J) treatment. One-way ANOVA analysis for (C) and (G). One asterisk (*) indicates *p* < 0.05, and 2 asterisks (**) indicate *p* < 0.01; n.s. indicates *p* > 0.05. All the original data can be found in S1 Data. FOH, farnesol; FPP, farnesyl pyrophosphate; GPP, geranyl pyrophosphate; TRPM2, transient receptor potential melastatin 2; WT, wild-type.

We next examined whether FPP could activate TRPM2 channel opening using whole-cell patch-clamp recording. In HEK-293T cells overexpressing TRPM2, the concentration of intracellular calcium was buffered to less than 1 nM by adding EGTA to the pipette solution, and currents were elicited by a voltage ramp lasting 250 ms from −100 mV to +100 mV (Fig 6A). We first verified the system with intracellularly application of adenosine diphosphate ribose (ADPR) (S5B and S5C Fig), showing that ADPR elicited obvious currents as reported [34]. Extracellular application of 12 μg/mL FPP activated the TRPM2 channels with an extended response time, and the current was significantly decreased after washout of FPP (Fig 6B and 6C). The FPP-induced current under these low intracellular calcium conditions were alleviated by the addition of TPPO (Fig 6D and 6E). As a critical control, GPP, which possessed 5 fewer carbons than FPP while having the same pyrophosphate head, and FOH, which had the same polyunsaturated hydrocarbon chain but not the pyrophosphate head compared to FPP, failed to activate TRPM2 channel (Fig 6F–6J). These results corresponded very well with the activities of FPP, GPP, and FOH in triggering acute cell death as described above (Fig 1B). Therefore, FPP is a novel danger signal that functions as a specific agonist of TRPM2 and results in acute cell death.

### FPP and TRPM2 signaling axis is involved in brain ischemia

The MVA pathway is highly active in the brain. About 25% of the total cholesterol in humans is found in the brain, and most of it is produced by local synthesis [35]. As a metabolic intermediate of the MVA pathway, FPP is ubiquitously distributed in different encephalic regions at variable concentrations [36]. In male patients with AD, the concentrations of FPP were found significantly elevated in both gray and white matter, compared with those in control cohorts [21]. Statins, which inhibit the synthesis of MVA and its downstream metabolites including cholesterol and FPP, not only decrease cholesterol levels but also reduce the incidence of ischemic stroke in both mice and humans [37]. How statins regulate brain ischemia remained unclear. Given that TRPM2 is most abundantly expressed in the brain [33] and that FPP-induced acute cell death is dependent on TRPM2 function, we sought to determine whether the FPP/TRPM2 signaling axis contributes to brain cell death in neurodegenerative diseases.

**Fig 7:**
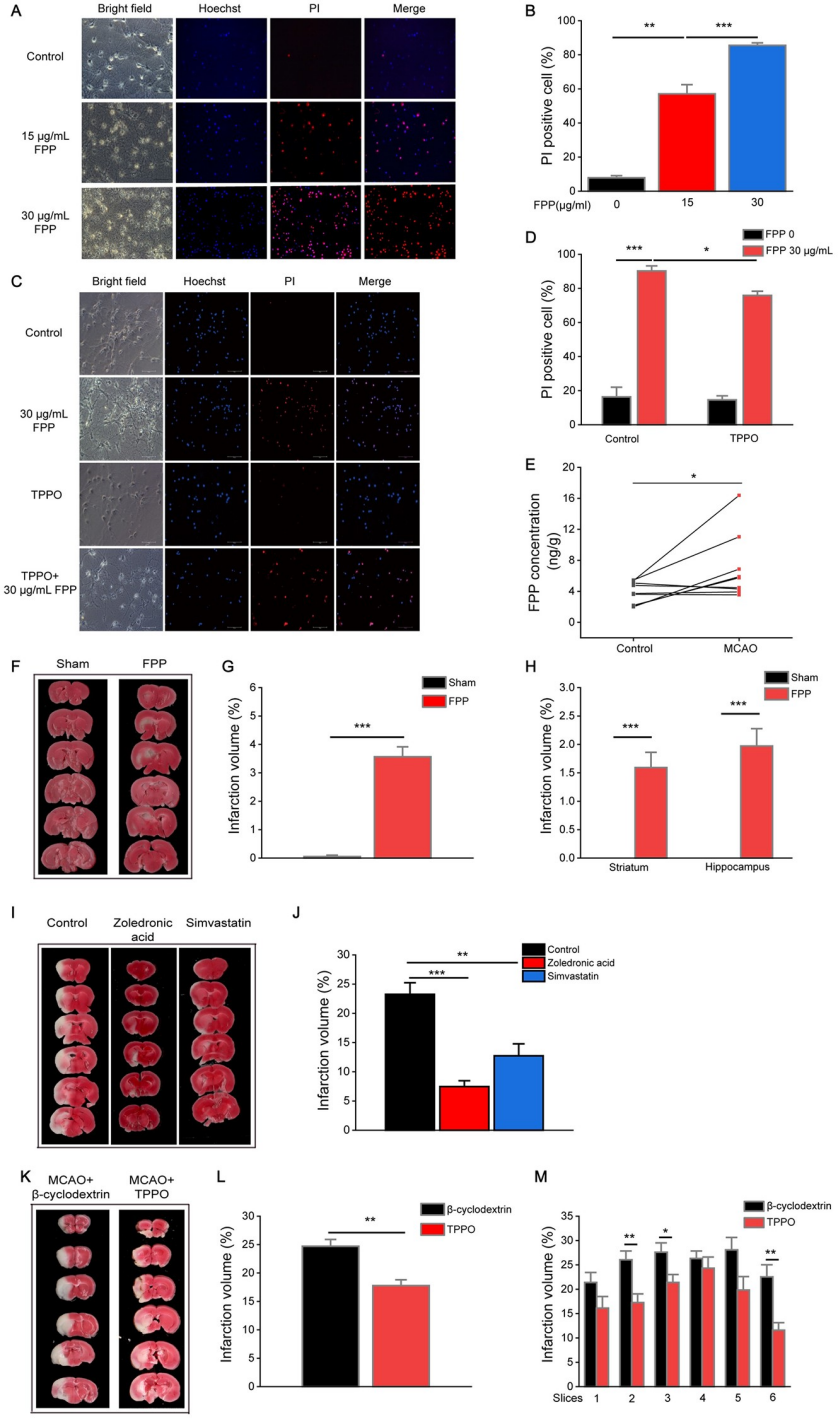
FPP accumulates in the brain of MCAO mice and FPP-TRPM2 axis is involved in mouse brain ischemic injury. (A) Neurons stained with Hoechst (blue) and PI (red). Bar denotes 100 μm. (B) Quantification of Hoechst–PI staining. Bars denote mean ± SEM. Nine fields from 3 independent experiments were evaluated. (C, D) TPPO effects on FPP-induced neuronal death. (C) Representative images under confocal microscopy, the scale bar is 100 μm. (D) Quantification of PI-positive cells versus Hoechst positive cells. Bars denote mean ± SEM. Nine fields from 3 independent experiments were evaluated. (E) FPP concentration in brains after 90 minutes MCAO and 24-hour reperfusion and brains without MCAO. One line means 1 mouse. (*n* = 9) (F) Representative images of serial coronal brain sections stained with TTC after FPP injection. (G, H) Statistical result of the whole brain infarction volume (G) or infarction volume of striatum and hippocampus (H) after FPP injection. Bars denote mean ± SEM. (*n* = 9 for FPP injection group and *n* = 6 for sham group) (I, J) Zoledronic Acid and Simvastatin decreased infarction volume in MCAO. A total of 3 μL Zoledronic Acid (10 mM) or Simvastatin (10 mM) were injected into the left striatum separately as we performed before, then the MCAO model was induced, and infarction volume were checked. (I) Representative images of serial coronal brain sections stained with TTC. (J) Quantification of whole brain infarct sizes. (*n* = 7 for Control; *n* = 7 for Zoledronic acid; *n* = 9 for Simvastatin). (K–M) TPPO decreases infarction volume in MCAO. Mice pretreated with TPPO and then the MCAO model was induced, and infarction volume were checked. (K) Representative images of serial coronal brain sections stained with TTC. (L) Quantification of whole brain infarct sizes or (M) infarction size of different slices of TTC-stained images. Bars denote mean ± SEM. (*n* = 6 for TPPO group and *n* = 9 for β-cyclodextrin group). One-way ANOVA analysis for Fig 7B, 7D, 7G, 7H, 7J, and 7K. Paired 2-tail *t* test for (E). One asterisk (*) indicates *p* < 0.05, 2 asterisks (**) indicate *p* < 0.01, and 3 asterisks (***) indicate *p* < 0.001. All the original data can be found in S1 Data. FPP, farnesyl pyrophosphate; MCAO, middle cerebral artery occlusion; PI, propidium iodide; TRPM2, transient receptor potential melastatin 2.

Using primary neuron culture, we found significant FPP-induced acute neuron death, similar to the acute cell death observed in FPP-treated P815 cells (Fig 7A and 7B). Pretreatment with TPPO alleviated primary neuron cell death induced by FPP (Fig 7C and 7D). In a mouse model of ischemic injury, MCAO, we also detected significant FPP accumulation (Fig 7E). With injection of exogenous FPP into the hippocampus and striatum of healthy mice, we induced cerebral infarction at the sites of injection, similar to that observed in ischemic injury (Fig 7F–7H). Conversely, transient application of HMG-CoA reductase inhibitor Simvastatin or the farnesyl diphosphate synthase (FPPS) inhibitor Zoledronic acid significantly decreased brain infarction volume in MCAO (Fig 7I and 7J). To further determine whether the FPP-induced TRPM2 activation is involved in neuronal death in vivo, we delivered the TRPM inhibitor TPPO via intrastriatal microinjection prior to the MCAO/reperfusion procedure. Compared to the vehicle control (β-cyclodextrin), TPPO exhibited a therapeutic effect of reducing the volume of cerebral infarction in the MCAO model (Fig 7K–7M).

Overall, our data demonstrate that FPP accumulates in the ischemic hemisphere and induces acute neuronal death in mouse brain ischemia and reperfusion. The infarct volume in brain ischemic injury can be alleviated by inhibiting this FPP-induced TRPM2-mediated cell death axis.

## Discussion

Our study discovered that FPP, a metabolic intermediate of the MVA pathway, could induce acute cell death in both cell lines and primary cells. The cytotoxic effect of FPP required both its lipophilic polyunsaturated hydrocarbon chain and the lipophobic pyrophosphate head. The acute cell death induced by FPP cannot be blocked by inhibitors acting downstream in the MVA metabolic pathway or inhibitors that were known to target the necroptosis pathway, small GTPases, and caspases. Extracellular calcium influx played a vital role in FPP-induced acute cell death, while some known ion channels including the orai1/2/3, potassium channels TREK1 and TRAAK, and P2RX channels were not involved. We further discovered that TRP channel TRPM2 played a significant role in mediating FPP-induced acute cell death. Remarkably, we identified that FPP was a novel agonist of TRPM2 and activate the channel opening of TRPM2. In vivo, FPP accumulates in MCAO model, and Simvastatin, Zoledronic acid, or TPPO treatment decreases the infarct volume in MCAO, suggesting the involvement of FPP and TRPM2 axis in the pathology of ischemia.

As a novel agonist of TRPM2, FPP has some structural similarities with the classical TRPM2 agonist ADPR. It has been reported that the ADPR-binding domain of TRPM2 is the intracellular NUDT9-H domain, which is a homolog of the human ADPR pyrophosphatase without the enzyme activity [38]. The pyrophosphate group and the terminal ribose group of ADPR are extremely vital for TRPM2 opening [39]. Considering the common pyrophosphate group (though with differences) shared by FPP and ADPR, it is likely that FPP activates TRPM2 in a way that anchors its lipophilic polyunsaturated hydrocarbon chain to the inner face of plasma membrane and reaches out its pyrophosphate head to the pocket of TRPM2, which binds the pyrophosphate of ADPR.

The FPP and TRPM2-mediated acute cell death is such a dramatic and toxic event that it is unlikely to happen under physiological conditions. We speculate that there might be at least 2 possible mechanisms to prevent FPP-induced cell death for physiological homeostasis. First, FPP is usually synthesized in mitochondria or in the cytosol together with FPP synthase, which is spatially isolated from the channels mainly expressed on the plasma membrane, such as TRPM2. Secondly, the lowest concentration of FPP that triggered acute cell death in our in vitro experiments is 9 μg/mL, which is much higher than the value reported in human plasma (0.25 ± 0.09 ng/mL and 1.61 ± 0.40 ng/mL in 2 independent studies) (S1 Table). Therefore, it is highly possible that FPP-induced cell death shall be expected to happen locally under extreme conditions such as ischemic injury when a large number of cells die and cannot be reached or diluted by the circulating blood. Although universally present in low concentration in human plasma, free FPP concentration in pancreatic cancer cell line lysate is much higher according to previous studies (S1 Table). Specifically, the measured concentration of FPP in AsPCl cell lysate is 0.84 ± 0.28 nmol per million cells, which equals to 4.65 mg/mL local FPP release after cell lysis, assuming the volume of 1 million red blood cell for an approximate calculation (S1 Table). Moreover, the concentration of FPP in other pancreatic cell line lysates including MiaPaca, BxPC3, Panc1, S2013, and Capan-1 shows a similar scale, although it is lower in NIH3T3 cell lysates (S1 Table). Taken together, it is possible that the amount of FPP released under extreme conditions could send a danger signal to the microenvironment and trigger severe cell death locally.

It has been reported that TRPM2 is involved in brain ischemic injury, attributing to its opening by ROS-induced production of ADPR [40,41]. This ischemic injury can be alleviated by TRPM2 inhibitor 2-APB and clotrimazole [40]. However, our data suggest that TRPM2 activation by FPP is different from its activation by ROS, as intracellular calcium release is not involved in TRPM2 activation by FPP while it is necessary for the activation by ADPR. And extracellular application of ADPR shows no cytotoxic effect (S5A Fig). Therefore, inhibitors that block TRPM2 activation by ROS such as clotrimazole, ketoconazole, or the ROS scavenger NAC cannot inhibit FPP-induced cell death. These results suggest that FPP is a novel danger signal that function via the activation of TRPM2 through a mechanism that is distinct from ROS-mediated TRPM2 activation. Given that clinical trials targeting oxidative stress to treat ischemic injury have failed [42], FPP activating TRPM2 and the downstream acute cell death may be novel druggable targets to treat ischemic injury. In view of the complex nature of human ischemic injury, targeting the FPP/TRPM2 axis may be combined with the current medicine to improve the therapeutic effects.

It is possible that the physiological consequence of FPP-induced cell death is similar to those of the cytolysis induced by ATP, although FPP and ATP function through different ion channels. FPP and ATP are produced universally in 2 different but related metabolic pathways. ATP is produced mainly by glycolysis and oxidative phosphorylation to supply energy for cellular events. However, under circumstances such as injury, inflammation, cancer, and immune cell activation, cell metabolic state shifts, and a larger portion of citrate is exported to the cytosol to be converted to Acetyl-CoA, which enables MVA metabolism and promotes the accumulation of isoprenoids [18]. Isoprenoid FPP is a key branch substrate for cholesterol synthesis, ubiquinones synthesis, protein farnesylation decoration, and GGPP synthesis [43]. When released under circumstances favoring its production, FPP may transduce signals to the surrounding tissues and cells, leading to cell death–associated pathogenesis. Our findings provide essential information which advances our knowledge and mechanistic understanding of the metabolic complexity of the cell in health and disease.

## Materials and methods

### Cells and reagents

P815, A20, and 293T cells were purchased from ATCC (Virginia, United States of America) and maintained in Roswell Park Memorial Institute (RPMI) 1640 Medium supplemented with 10% FBS, MEM Non-Essential Amino Acids Solution (Gibco, Shanghai, China, #11140050), Penicillin-Streptomycin (Gibco, #15140122), and Sodium Pyruvate (Gibco, #11360070). Spleen cells and thymocytes were acquired by milling the spleen or thymus from C57BL/6 or other genotyped mice as indicated. Red blood cells were lysed using RBC lysis buffer (10X) from BioLegend (Santiago, USA) and maintained in RPMI-1640 medium supplemented with 5 μg/mL lipopolysaccharides (LPS, #L4391) from Sigma (Shanghai, China) until use. Moreover, 293T cells were maintained in DMEM medium (#10569010) and digested using 0.05% Trypsin-EDTA (Gibco, #25200056) when necessary. STIM1 knockout and IP3R1/2/3 knockout DT40 cells were from Dr. Tomohiro Kurosaki and Dr. Hisaaki Shinohara (WPI Immunology Frontier Research Center, Osaka University, Japan). Saline buffer was purchased from Zhongkekeao (Beijing, China) (#R00641) and HBSS buffer without calcium and magnesium from Gibco (#14170112). PI was acquired from Sigma (#P4170) and reconstituted with water to final concentration of 5 mg/mL. All the salt reagents were from Guoyao (Beijing, China) except CaCl_2_ (#449709), MgCl_2_ (#449172), BaCl_2_ (#449644), and SrCl_2_ (#451282) from Sigma. FPP analogs FOH was purchased from Sigma (#F203) and FSPP from Echelon Bioscience (Salt Lake City, USA, #I-S150). The information of all the dyes used in the experiment is as follows: Hoechst 33342 (Invitrogen, Shanghai, China, #H1399), fluo-4 AM (Invitrogen, #F14201), Mitosox red mitochondrial superoxide indicator (Yeasen, Beijing, China, #40778), and jc-1 (Leagene, Beijing, China, #CT0045).

### Inhibitors

GGTI2133 was bought from Santa Cruz Biotechnology (Dallas, USA, #1217480-14-2). FTI was from Selleck (Texas, USA, #S7465). CsA was from Selleck (#S2286). NAC was from Sigma (#A7250). Necrosulfonamide (NSA, # 432531) and Necrostatin-1 (Nec-1) (Sigma #480065) were from Sigma. Nec-1s (VWR, Pennsylvania, USA, #852391-15-2), zvad-fmk (Sigma #V116), CT04 (Cytoskeleton, Denver, USA, #CT04), Ehop016 (Selleck #S7319). During the experiments, cells were pretreated with inhibitors for 30 minutes and then went for following stimulations.

### Cell line construction

The general process of doing gene knockout in P815 cells was first cloning the sgRNA sequence into the lenti-puro (Addgene, Massachusetts, USA, #52963) backbone and co-transfecting it with the psPAX2 and pMD2.G to HEK-293T cells to produce viruses containing target sgRNAs. Moreover, 48 hours after the transfection, viruses were harvested, filtered, and infected P815 cells for another 48 hours in the presence of polybrene. The sgRNA positive cells were enriched by 6 μg/mL puromycin selection. For knockout in A20 cells, sgRNAs were cloned into the Bbs1 digested pSpCas9-2a-GFP plasmid by T4 ligase. The constructed plasmids were amplified, extracted, and transfected into A20 cells through electroporation. Two days after the electroporation, high GFP expression cells were enriched by flow cytometry selection. All the primers used for cell line construction were listed in S2 Table. For Bcl2 overexpression cell line construction, plasmids Bcl2-IRES-mcherry and control mcherry were transfected into P815 cells using the lentivirus infection system as indicated above, and the mcherry positives were selected out by flow cytometry.

### PI signal intensity change assay

For experiments using P815 cells, cells were washed 2 times with PBS and resuspended with Tyrode’s buffer (137 mM NaCl, 2.7 mM KCl, 1.0 mM MgCl_2_, 1.8 mM CaCl_2_, 20 mM HEPES, and 5.6 mM glucose, pH 7.4) or saline buffer or HBSS without calcium and magnesium to final concentration of 10E5 cells per 80 μL. For FPP-induced cell death in Tyrode’s buffer, 20 μL FPP in saline of indicated concentrations were added into cells seeded in black 96-well plate (Corning, New York, USA, #3925) and mixed. Fluorescence of each well under the excitation of 531 nm and emission of 632 nm was read before FPP addition every 30 seconds for 10 minutes and after FPP addition for 60 minutes using VARIOSKAN FLASH microplate reader. Fluorescence data was normalized according to the value before FPP addition, and the results were presented using GraphPad software. For experiments doing in saline buffer or HBSS without calcium or magnesium, slight modifications were made as after FPP addition cells were allowed to react for 10 minutes and then CaCl_2_ was added into each well to 2 mM working concentration to promote the reaction. For other cells, cell number was adjusted according to experiments. For all the experiments involving inhibitors, 20 to 30 minutes was taken for inhibitors fully interacting with cells before adding FPP.

For cell death measurement after FPP treatment by flow cytometry, cells were treated in the same way as indicated above; after 1-hour incubation, PI signal was measured by LSR (BD Biosciences, California, USA), and the results were analyzed by Flowjo software.

### Neural cell death assay

Hippocampal neurons were obtained from Sprague-Dawley rats at embryonic day 18. Briefly, hippocampi were isolated and digested with a 0.25% Trypsin solution for 30 minutes at 37 °C. After that, a Trypsin inactivation solution containing 5% fetal bovine serum (Gibco) was applied. The neurons were cultured onto poly-L-lysine (Sigma-Aldrich, Shanghai, China)-coated 18-mm coverslips in 12-well culture dishes at a density of 120,000 cells per well. Wells contained the glia-conditioned neurobasal media (Gibco) supplemented with 2% B-27 (Gibco), 2 mM Glutamax (Gibco), and 1% penicillin-streptomycin (Gibco). FDU was added to the media at DIV 3 to inhibit the growth of glial cells.

After 10 days in vitro, hippocampal neurons were stained with Hoechst 33342 and PI (Sigma-Aldrich). Briefly, cells were incubated with media containing Hoechst (5 μg/mL) at 37 °C for 30 minutes. Then, the cells were washed with ACSF (HEPES (pH7.4, 1 M) 25 mM, NaCl (5 M) 125 mM, KCl (1 M) 2.5 mM, Glucose (1 M) 30 mM, MgCl_2_ (1 M) 1 mM) twice. After that, ACSF containing PI (5 μg/mL) was added to each well and incubated for 10 minutes and then CaCl_2_ was added to 2 mM working concentration to promote the reaction. The coverslips were photographed with a fluorescence microscope (Leica, Illinois, USA). Results were quantified with ImageJ software and expressed as the percentages of PI-positive cell.

### Cell Counting Kit-8 assay

CCK8 kit (Sigma, #96992) was used to check the viability of cells after FPP treatment according to the standard protocol. Briefly, P815 cells suspended in Tyrode’s buffer were aliquoted into 96-well plate and treated with different concentration of FPP at 37 °C with 5% CO_2_ for 1 hour. After that, the plate was centrifuged, and supernatants were aspirated into a new plate. A total of 10 μL of the CCK-8 solution was added to each well, and the plate was incubated at 37 °C for 2 hours before reading the absorbance at 450 nm using the microplate reader.

### Calcium mobilization by flow cytometry

To measure calcium influx after FPP treatment, cells were stained with 2 μg/mL fluo-4 AM in the presence of 2 μg/mL probenecid (Invitrogen,#P36400) in HBSS for 30 minutes at 37 °C, washed with HBSS for 1 time, and incubated for another 15 minutes. Then cells were washed and resuspended in saline buffer. FPP was added to react for 5 minutes, and the baseline fluorescence was read through BD Accuri C6 Plus for 30 seconds. Then 2 mM CaCl_2_ was added, and the calcium influx within 4 minutes was detected. For the control group, no CaCl_2_ was added.

### Calcium mobilization and cell death imaging by confocal microscopy

To measure calcium influx after FPP treatment through confocal microscopy, cells were stained with fluo-4 AM in the same way as indicated above and resuspended in saline buffer in the presence of 2.5 μg/mL PI and FPP. Then cells were seeded into chambers ready for confocal imaging (Olympus, Tokyo, Japan, 40X scope). Images were captured every 30 seconds for around 2 minutes, and CaCl_2_ was added to monitor calcium influx.

### Mitochondrial ROS and potential measurement

Mitochondrial ROS and potential were measured in accordance with the standard protocol. Briefly, cells were stained with 5 μM MitoSox for 10 minutes at 37 °C in HBSS, then washed with PBS and resuspended in saline. FPP was added and incubated for 5 minutes at room temperature (RT). After that, mitosox signal was checked using BD Accuri C6 Plus (California, USA). For the group with calcium, 2 mM CaCl_2_ was added and incubated for another 5 minutes before flow measurement. For mitochondrial potential detection, cells were stained with 2 μM jc-1 at 37 °C in HBSS for 15 minutes, washed, and resuspended in saline. FPP treatment and flow cytometry measurements were consistent with the conditions above.

### RNA isolation and qPCR

Total RNA was extracted using HiPure total RNA mini kit (Magen, Guangzhou, China, #R4111) according to the standard protocol. RNA was reversely transcribed to cDNA using RevertAid RT Reverse Transcription Kit (Thermo Fisher Scientific, Shanghai, China, #K1691) with polyA primers. Quantitative PCR (qPCR) was performed using TB Green Premix Ex Taq II (Tli RNase H Plus) (Takara, Beijing, China, #RR802B). All the primer sequences were presented in S2 Table.

### Patch clamp

Patch clamp was performed in the whole-cell configuration (HEKA EPC10) at RT. Data were digitized at 20 kHz. Patch pipettes were pulled from borosilicate glass to a resistance of 3 to 8 MΩ when filled with internal solution. Series resistance (Rs) was compensated up to 70% at least. A voltage ramp lasting 250 ms from −100 mV to +100 mV were delivered from a holding potential of 0 mV. 293T cells were kept in the standard extracellular Ringer’s solution containing (in mM) 140 NaCl, 2.8 KCl, 2 MgCl_2_, 10 glucose, and 10 HEPES (pH 7.2, osmolarity 298 to 308 mOsm). The internal pipette solution for TRPM2 channel recording contained (in mM) 145 Cs-methanesulfonate (CsSO_3_CH_3_), 8 NaCl, 1 EGTA, and 10 HEPES (pH 7.2 adjusted with CsOH, osmolarity 295 to 305 mOsm). Free [Ca^2+^]_i_ was calculated with WEBMAXC (https://somapp.ucdmc.ucdavis.edu/pharmacology/bers/maxchelator/webmaxc/webmaxcS.htm). TPPO (50 μM) was added to bath solution before patch-clamp experiments to pretreat the cells for 15 minutes. FPP (12 μg/mL) was added to bath solution to detect the effect of FPP on TRPM2.

### Animals

Adult C57BL/6J mice (male, 10 to 12 weeks old, weight: 22 to 26 g) were used in the present study. All animals were reared in a temperature- and humidity-controlled room (temperature was kept at 24 to 25 °C and humidity was kept at 50% to 60%) and given ad libitum access to standard chow and water under 12-hour light/dark cycle. All animal experiments were approved by the Animal Research Ethics Committee (Approval Number: LA2020233), Peking University and were performed in accordance with the Animal Management Rules of Ministry of Health of the People’s Republic of China.

### Mouse model of middle cerebral artery occlusion and TTC staining

Transient focal cerebral ischemia (90 minutes) was induced by MCAO through the intraluminal filament methods. Briefly, mice were deeply anesthetized with 1% sodium pentobarbital intraperitoneally at a dose of 70 mg/kg. An incision was made at the middle of the neck, and the left carotid artery was separated carefully. Then a filament suture was inserted into the internal carotid artery until small resistance was felt. Mice were kept at 37 °C during the surgery with a heating pad. The filament suture was removed carefully 90 minutes after occlusion and the incision was sutured.

After 24-hour reperfusion, mice were humanely killed, and the brains were harvested for the TTC staining. In brief, the brains were sectioned into 1-mm thick slices using the brain matrix.

Then the slices were immersed for 30 minutes into 1% TTC solution at RT. Mice with hemorrhage in the skull base were excluded from the assay. The stained slices were photographed with digital camera. The infract area of each slices was measured by ImageJ software.

### Brain FPP injection

Mice were anesthetized with sodium pentobarbital (70 mg/kg, i.p.) and fixed on the stereotaxic apparatus. Two small holes were drilled in skull using a dental drill for microinjection. To investigate the effects of FPP on the mice brain in vivo, 3 μL FPP (30 mg/mL) or 0.9% NaCl (solvent control) was injected into the left striatum (anterior/posterior, +0.5 mm; medial/lateral, −2.0 mm; dorsal/ventral −2.5 mm according to the mouse brain atlas) and left hippocampus (anterior/posterior, −1.7 mm; medial/lateral, −1.0 mm; dorsal/ventral −1.5 mm) via a Hamilton microsyringe at the rate of 0.3 μL/min. The microsyringe was left in place for an additional 5 minutes before withdrawal. After 24 hours, the mice were humanely killed, and the brains were removed for TTC staining.

### Intrastriatal TPPO microinjection

TPPO was dissolved in β-cyclodextrin at a concentration of 1 mg/mL with a quality ratio of 1:25. Mice were anesthetized with sodium pentobarbital (70 mg/kg, i.p.) and fixed on the stereotaxic apparatus. A small hole was drilled in skull using a dental drill for microinjection. To investigate the effects of TPPO on the infract area of mice, 3 μL TPPO (1 mg/mL) or β-cyclodextrin solution (solvent control) was injected into the left striatum (anterior/posterior, +0.5 mm; medial/lateral, −2.0 mm; dorsal/ventral −2.5 mm according to the mouse brain atlas) via a Hamilton microsyringe at the rate of 0.3 μL/min. The microsyringe was left in place for an additional 5 minutes. MCAO was performed after the microinjection. After the mice were humanely killed, the brains were removed for TTC staining. The proportion of infarct areas of each brain sections were measured by ImageJ and analyzed with originlab. Two-tailed *t* test was performed between the TPPO treated group and the control group to calculate the differences.

### Brain FPP concentration measurement

Both the left and right brains from mice after MCAO were harvested, and the olfactory bulb and cerebellum were cut and abandoned. The wet weight of the rest tissues was measured and recorded. Then FPP was extracted adopted from the protocol published by Henneman and colleagues [44]. In short, tissues were homogenized in 500 μL 2-propanol:100 mM NH_4_HCO_3_ pH 7.8 (1:1 v/v), and 500 μL acetonitrile was added. After incubating on ice for 10 minutes, the mix was centrifuged at 14,000 g for 10 minutes, and the supernatants were aspirated and spun drying. The pellets were reconstituted in 50 μL methanol, and 1 μL was get out for measuring FPP concentration using triple quadrupole in the Center of Pharmaceutical Technology in Tsinghua University. The standard curve was drawn using standard synthesized FPP. Representative FPP peaks under triple quadrupole mass spectrometry were shown in S6 Fig.

## Supporting information

S1 Text Supplementary chemical synthesis. The methods of synthesizing IPP-GGPP, FPP analogs, BPH-652, BPH-754, and GGT2-I. FPP, farnesyl pyrophosphate; GGPP, geranyl-geranyl pyrophosphate; IPP, isopentenyl pyrophosphate.
(DOCX)

S1 Fig (A, B) Bax/Bak KO in BMDM did not affect FPP-induced cell death. Cre-IRES-mcherry were transfected into WT or Bax/Bak flox BMDM, then treated with 100 μg/mL FPP in the presence of Ca^2+^ for 1h and check the mcherry positive cell number change. (A) showed the representative images and (B) showed the statistical results. The scale bar is 100 μm. (C, D) Bcl2 overexpression did not affect FPP-induced cell death. Bcl2-IRES-mcherry and control mcherry was transfected into P815 cells. The mcherry positivity of Bcl2 group is 6% higher than that of control group. After being treated with FPP for 40 minutes, the mcherry fluorescence were checked with flow cytometry, and mcherry negative cells were counted as dead cells. Unpaired *t* test is used, and n.s. indicates *p* > 0.05. The original data in this figure except S1C Fig can be found in S2 Data. The original data in S1C Fig can be found in S5 Data. BMDM, bone marrow–derived macrophage; FPP, farnesyl pyrophosphate; KO, knockout; WT, wild-type.
(TIF)

S2 Fig Conventional cell death pathway, caspase and 2 small GTPase inhibitors cannot suppress FPP-induced cell death. (A) Table with different inhibitors used, their functions, and their effects on FPP-induced cell death. (B) Effects of inhibitors targeting conventional cell death pathway, caspase, and GTPase on FPP-induced cell death in P815 cells. Significant difference between 50 μM Nec-1s FPP and 0 Nec1s FPP shows up from 27 minutes on (*p* < 0.05). Significant difference between 100 μM Nec-1s FPP and 0 Nec1s FPP shows up from 23 minutes on (*p* < 0.05). Significant difference between 20/40 μM Zvad-fmk FPP and 0 Zvad-fmk FPP shows up from 30 minutes on (*p* < 0.05). All the other inhibitors treatments show no significant differences compared to the corresponding controls (*p* > 0.05). Two-way ANOVA analysis is used. (C) GSDMD KO cannot significantly inhibit FPP-induced cell death. Spleen cells from WT and GSDMD KO mice were cultured in the presence of LPS. Moreover, 24 hours later, cells were treated with different concentrations of FPP, and the PI signal changes were measured. Each treatment had 3 to 4 replicates. Bars denote mean ± SEM (SEM in gray). Two-way ANOVA analysis is used, and no significant differences are observed between the experimental and corresponding control (*p* > 0.05). All the original data can be found in S2 Data. FPP, farnesyl pyrophosphate; KO, knockout; PI, propidium iodide; WT, wild-type.
(TIF)

S3 Fig Extracellular Ca^2+^, Ba^2+^, and Sr^2+^ lead to acute cell death after FPP treatment, while intracellular calcium depletion is not necessary. (A, B) FPP-induced cell death in the presence of different cations including Ca2+, Mg2+, Sr2+, and Ba2+ at 2 different concentrations, (A) 0.5 mM and (B) 2 mM. Significant difference between 30 μg/mL FPP 0.5 mM Ca2+ and the control shows up from 5 minutes on (*p* < 0.05). Significant difference between 30 μg/mL FPP 0.5 mM Ba2+ and the control shows up from 17 minutes on (*p* < 0.05). Significant difference between 30 μg/mL FPP 2 mM Ca2+ and the control shows up from 5 minutes on (*p* < 0.05). Significant difference between 30 μg/mL FPP 2 mM Ba2+ and the control shows up from 8 minutes on (*p* < 0.05). All the others show no significant difference (*p* > 0.05). (C) Effects of ER calcium depletion inhibitor 2-APB on FPP-induced cell death in P815 cells. No significant differences are observed between the 2-APB treated and untreated group (*p* > 0.05). (D) 2-APB effects on calcium influx after DT40 cells were stimulated with anti-IgG(H+L). (E) PI signal changes after FPP treatment in control cells and IP3R1/2/3 KO and STIM1 KO DT40 cells. Black arrows indicate the time points at which different reagents were added. Bars denote mean ± SEM (SEM in gray). No significant differences are observed between the KO and WT cells (*p* > 0.05). Two-way ANOVA analysis is used. All the original data can be found in S2 Data. ER, endoplasmic reticulum; FPP, farnesyl pyrophosphate; KO, knockout; PI, propidium iodide; WT, wild-type.
(TIF)

S4 Fig Two pore potassium channels, Orai channels and P2X channels, are not involved in FPP-induced cell death. (A) PI signal changes after FPP treatment in control cells and ORAI1/2/3/ KO cells. Significant difference between Orai2 KO FPP and the control shows up from 15 minutes on (*p* < 0.05). All the others show no significant difference (*p* > 0.05). (B) PI signal changes after FPP treatment in control cells and TREK1/TRAAK KO cells. No significant differences are observed between the KO and WT cells (*p* > 0.05). (C) PI signal changes after FPP treatment in control cells and P2RX1/4/5/6/7 KO cells. Significant difference between P2RX5 KO FPP and the control shows up from 44 minutes on (*p* < 0.05). All the others show no significant difference (*p* > 0.05). (D) PI signal changes after FPP treatment in the presence of P2RX7 blocker brilliant blue G. Black arrows mean the time points at which different reagents are adding. Bars denote mean ± SEM (SEM in gray). No significant difference is observed between the inhibitor treated and untreated group (*p* > 0.05). Same controls used in Orai1 KO, Orai2 KO, P2RX5 KO, and P2RX7 KO because that are from 1 experiment. Data are representative of at least 2 independent experiments in (A) to (D). Two-way ANOVA analysis is used. All the original data can be found in S2 Data. FPP, farnesyl pyrophosphate; KO, knockout; PI, propidium iodide; WT, wild-type.
(TIF)

S5 Fig Extracellular ADPR is not cytotoxic though intracellular ADPR activate TRPM2. (A) ADPR cannot lead to acute cell death. P815 cells were resuspended in SLH buffer, and different concentrations of ADPR and 2 mM CaCl_2_ were added sequentially. PI signal intensity was measured during the process. Each treatment had 3 to 4 replicates. Bars denote mean ± SEM (SEM in gray). The first black arrow indicates the addition of ADPR, and the second black arrow indicates the addition of CaCl_2_. Two-way ANOVA analysis is used and no significant differences are observed between the ADPR treated and non-treated group (*p* > 0.05). (B) ADPR elicit TRPM2 current in TRPM2 overexpressed 293T cells. Current change of TRPM2 channels after intracellular addition of 200 μM ADPR (ADPR) or the control intracellular buffer without ADPR (Control). (*N* = 16 for Control; *N* = 16 for ADPR) This experiment is done according to the method published by Du et al. [34]. (C) The representative TRPM2 current trace elicited by the voltage ramp after ADPR stimulation. All the original data can be found in S2 Data. ADPR, adenosine diphosphate ribose; PI, propidium iodide; TRPM2, transient receptor potential melastatin 2.
(TIF)

S6 Fig Representative results of FPP measurement under triple quadrupole mass spectrometry. FPP, farnesyl pyrophosphate.
(TIF)

S1 Table FPP concentration from published papers. FPP, farnesyl pyrophosphate.
(XLSX)

S2 Table Primers for cell line construction and qPCR. qPCR, quantitative PCR.
(XLSX)

S1 Data Raw data used for all figures from Figs 1 to 7. (XLSX)

S2 Data Raw data used for all figures from S1 to S5 Figs. (XLSX)

S3 Data Raw data for FCS files in Figs 1D, 3C and 3D and 4E. (ZIP)

S4 Data Raw data for FCS files in Fig 4F. (ZIPX)

S5 Data Raw data for FCS files in S1C Fig. (ZIPX)

## Abbreviations

AD: Alzheimer disease
ADPR: adenosine diphosphate ribose
BCR: B cell receptor
BMDM: bone marrow–derived macrophage
CCK8: Cell Counting Kit-8
CsA: cyclosporin A
DMAPP: dimethylallyl pyrophosphate
ER: endoplasmic reticulum
FOH: farnesol
FPP: farnesyl pyrophosphate
FPPS: farnesyl diphosphate synthase
FSPP: farnesyl S-thiolodiphosphate
FTI: farnesyl transferase inhibitor
GGPP: geranyl-geranyl pyrophosphate
GPP: geranyl pyrophosphate
HMGB1: high mobility group protein B1
IP3R: inositol trisphosphate receptor
IPP: isopentenyl pyrophosphate
LPS: lipopolysaccharides
MCAO: middle cerebral artery occlusion
MVA: mevalonate
NAC: N-acetyl-l-cysteine
Nec-1: necrostatin-1
NSA: necrosulfonamide
PI: propidium iodide
qPCR: quantitative PCR
ROS: reactive oxygen species
RPMI: Roswell Park Memorial Institute
RT: room temperature
STIM1: stromal-interacting molecule 1
TRP: transient receptor potential
TRPM: transient receptor potential melastatin
TRPM2: transient receptor potential melastatin 2
