# Correction: Farnesyl pyrophosphate is a new danger signal inducing acute cell death

**DOI:** 10.1371/journal.pbio.3002414

**Published:** 2023-11-15

**Authors:** Jing Chen, Xiaochen Zhang, Liping Li, Xianqiang Ma, Chunxiao Yang, Zhaodi Liu, Chenyang Li, Maria J. Fernandez-Cabezudo, Basel K. al-Ramadi, Chuan Wu, Weishan Huang, Yong Zhang, Yonghui Zhang, Wanli Liu

The following information is missing from the Funding statement:

W.L. was also supported by grants from National Natural Science Foundation of China (81825010), and Ministry of Science and Technology of China Grants (2021YFC2300500 and 2021YFC2302403).
