# Supplementary material for: Farnesyl pyrophosphate is a new danger signal inducing acute cell death
Source: PLoS Biol. 2021 Apr 26;19(4):e3001134. doi: 10.1371/journal.pbio.3001134 (PMC8075202; doi:10.1371/journal.pbio.3001134)
Supplement: S1 Text — The methods of synthesizing IPP-GGPP, FPP analogs, BPH-652, BPH-754, and GGT2-I. FPP, farnesyl pyrophosphate; GGPP, geranyl-geranyl pyrophosphate; IPP, isopentenyl pyrophosphate. (DOCX) [file pbio.3001134.s001.docx]

**S1 Text. Supplementary chemical synthesis. The methods of synthesizing IPP-GGPP, FPP analogs, BPH-652, BPH-754 and GGT2-I.**

**General synthetic approach to isoprenoid diphosphates (IPP - GGPP)**

The main synthetic steps of isoprenoid diphosphates were carried out based on a previous report [1]. In brief, they were prepared from corresponding isoprenyl chloride or methylsulfonate, as outlined in **Scheme 1*^a^***.

**Scheme 1*^a^***

*^a^*Reagents and conditions: (a) Et_3_N, methanesulfonic anhydride, CH_2_Cl_2_; (b) tris(tetra-*n*-butylammonium) hydrogen pyrophosphate, MeCN; (c) N-Chlorosuccinimide, Me_2_S, CH_2_Cl_2_.

1. **Methyl-3-buten-1-yl diphosphate (IPP)**

To a solution of 3-methyl-3-buten-1-ol (**1**) (86 mg, 1.0 mmol) in dry CH_2_Cl_2_ was added methanesulfonic anhydride (209 mg, 1.1 mmol) and DMAP (244 mg, 2.0 mmol), and then the mixture was stirred for 2 hours. The resulting solution was poured into water and extracted with CH_2_Cl_2_. The combined organic layers were dried over MgSO_4_, filtered and concentrated under reduced pressure to provide 3-methyl-3-buten-l-yl methylsulfonate ester (**2**), which could be used for the next step without further purification. Then a solution **2** (160 mg, 1.0 mmol) in acetonitrile was added to a stirred solution of tris (tetra-n-butylammonium) hydrogen pyrophosphate (1.81 g, 2.0 mmol) in 3.5 mL of acetonitrile. The resulting solution was stirred for 2 h and converted to the ammonium form by using Dowex (NH4^+^). The solution was lyophilized to afford a solid, which was dissolved in aqueous NH_4_HCO_3_ (2 mL, 0.1 M) and washed three times with 5 mL 1:1 (v/v) MeCN/i-PrOH. Lyophilization finally yielded a white powder (147 mg, 60%). HRMS calc. 245.0023; found 245.0029. ^1^H NMR (400 MHz, D_2_O) δ ppm 4.85 (d, *J* = 9.88 Hz, 2H), 4.07 (dd, *J_1_* = *J_2_* = 6.64 Hz, 2H), 2.41 (t, *J* = 6.40 Hz, 2H), 1.79 (s, 3H). ^31^P NMR (162 MHz, D_2_O) δ ppm -10.73 (d, *J* = 17.82 Hz, P1), -8.74 (d, *J* = 17.82 Hz, P2).

**4-Methyl-2-buten-1-y1 diphosphate (DMAPP)**

The synthesis of DMAPP was similar to that of IPP synthesis, in which a direct displacement occurred at the activated position of 3-methyl-2-buten-1-yl bromide (**3**) by tris (tetra-n-butylammonium) hydrogen pyrophosphate. HRMS calc. 245.0021; found 245.0027. ^1^H NMR (400MHz, D_2_O) δ ppm 5.44 (t, *J* = 6.8 Hz, 1H), 4.44 (t, *J_1_* = *J_2_* = 6.72 Hz, 2H), 1.75 (s, 3H), 1.71 (s, 3H). ^31^P NMR (162 MHz, D_2_O) δ ppm -7.38 (d, *J* = 21.55 Hz, P1), -10.39 (d, *J* = 21.55 Hz, P2).

**(*E*)-3,7-dimethyl-2,6-octadien-l-y1** **diphosphate (GPP)**

To a solution of N-chlorosuccinimide (150 mg, 1.1 mmol) in dry CH_2_Cl_2_ was added dimethyl sulfide (0.08 mL, 1.2 mmol) dropwise under nitrogen at -40^o^C. Then the mixture was warmed to 0^o^C and stirred for 5 minutes. Subsequently, (*E*)-3, 7-dimethylocta-2,6-dien-1-ol (**4**, 154 mg, 1 mmol) dissolve in dry CH_2_Cl_2_ (5 mL) was added to the solution at -40^o^C. The mixture was slowly allowed to warm to 0^o^C over 1 hour and maintained at 0^o^C for another hour, after which time it was then warmed to room temperature for 15 min. Then pouring the solution into a 100 mL separatory funnel containing 30 mL cold brine solution. The aqueous phase was extracted with hexanes. The combined organic layers were washed with cold brine and dried over anhydrous MgSO_4_ and the solvent was then removed to give crude product (**7**), which could be used for the next step without further purification. GPP, as a white solid (110 mg, 35%), was synthesized in a similar method of the last step of IPP synthesis, from **7**. HRMS calc. 313.0603; found 313.0609. ^1^H NMR (400 MHz, D_2_O) δ ppm 5.45 (t, *J* = 6.64 Hz, 1H), 5.20 (t, *J* = 5.40 Hz, 1H), 4.47 (dd, *J_1_* = *J_2_* = 7.4 Hz, 2H), 2.11 (m, 4H), 1.72 (s, 3H), 1.68 (s, 3H), 1.62 (s, 3H). ^31^P NMR (162 MHz, D_2_O) δ ppm -10.61 (d, *J* = 19.44 Hz, P1), -9.42 (d, *J* = 19.44 Hz, P2).

(***E, E*)-3,7,11-trimethyl-2,6,10-dodecatrien-** **1-yl diphosphate** **(FPP)**

FPP was prepared from (*E, E*)-3,7,11-Trimethyl-2,6,10-dodecatrien-1-ol (**5**), according to the synthesis of GPP, as a white solid (152 mg, 40% yield). HRMS calc. 381.1207; found 381.1209. ^1^H NMR (400 MHz, D_2_O) δ ppm 5.46 (t, *J* = 6.6 Hz, 1H), 5.20 (m, 2H), 4.47 (t, *J_1_* = *J_2_* = 6.64 Hz, 2H), 2.16-2.02 (m, 8H), 1.71 (s, 3H), 1.68 (s, 3H),1.61 (s, 3H). ^31^P NMR (162 MHz, D_2_O) δ ppm -10.60 (d, *J* = 21.06 Hz, P1), -9.41 (d, *J* = 21.06 Hz, P2).

**(*E, E, E*)-3,7,11,15-tetramethylhexadeca-2,6,10,14-tetraen-1**-**yl** **diphosphate** **(GGPP)**

The initial five steps of GGPP synthesis is reported by Roe, S.J [2]. The last step, using (*E,E,E*)-3,7,11,15-tetramethylhexadeca-2,6,10,14-tetraen-1-ol (**6**), was synthesized (40% yield) in a similar manner to that of GPP. HRMS calc. 449.1903; found 449.1909. ^1^H NMR (400 MHz, D2O) δ ppm 5.48 (t, *J* = 7.0 Hz, 1H), 5.21-5.17 (m, 3H), 4.49 (t, *J_1_* = *J_2_* = 6.20 Hz, 2H), 2.11-2.03 (m, 12H), 1.75 (s, 3H), 1.69 (s, 3H), 1.64 (s, 3H), 1.62 (s, 3H). ^31^P NMR (162 MHz, D2O) δ ppm -10.72 (d, *J* = 21.06 Hz, P1), -7.89 (d, *J* = 21.06 Hz, P2).

**General synthetic approach to FPP analogs.**

The synthetic methods of FPP analogs (**FPP Mu1-4**) are shown in **Scheme 2*^a^***.

**Scheme 2^a^**

*^a^* Reagents and conditions: (a) 1-bromohexane, K_2_CO_3_, acetone, 60^o^C, 12 h; (b) LiAlH_4_, THF, 0^o^C, 1 h; (c) N-Chlorosuccinimide, Me_2_S, CH_2_Cl_2_ (dry), -40^o^C - 0^o^C, 2 h; (d,e) tris(tetra-*n*-butylammonium) hydrogen pyrophosphate, MeCN; (f) tris (tetra-*n*-butylammonium) hydrogen ethylenediphosphonate, MeCN; (g) tris (tetra-*n*-butylammonium) hydrogen hydroxyethanediphosphonate, MeCN.

1. **(Hexyloxy)pyridin-3-yl)methyl trihydrogen diphosphate (FPP-Mu1)**

A solution of methyl 5-hydroxynicotinate (**10**, 310 mg, 2.0 mmol), 1-bromohexane (400 mg, 2.4 mmol) and K_2_CO_3_ (550 mg, 4.0 mmol) in acetone was stirred overnight at 60^o^C. Then the mixture was filtered and concentrated to give crude **11**, which was used for the next step without further purification. To a slurry of LiAlH_4_ (110 mg) in THF was added dropwise a solution of **11** in THF under N_2_ at 0^o^C. After 1 hour, water (1 mL) was added slowly to quench the reaction. The mixture was filtered, concentrated and purified by flash chromatography to yield **12**. Then FPP-1 was prepared from intermediate **12**, and **13**, using a procedure similar to that of GPP, as a white solid (129 mg, 35%). HRMS calc. 420.1569; found 420.1565. ^1^H NMR (400 MHz, CDCl_3_) δ ppm 8.14 (s, 1H), 8.07 (s, 1H), 7.48 (s, 1H), 4.97 (d, *J* = 6.46 Hz, 2H), 4.05 (s, 2H), 1.71 (d, *J* = 6.50 Hz, 2H), 1.39 (s, 2H), 1.28 (s, 4H), 0.84 (s, 3H). ^31^P NMR (162 MHz, D_2_O) δ ppm -8.18 (d, *J* = 21.11 Hz, P1), -10.90 (d, *J* = 21.11 Hz, P2).

**3,7,11-Trimethyldodecyl trihydrogen diphosphate (FPP-Mu2)**

FPP-Mu2 was synthesized from the reaction of **14** (228 mg, 1.0 mmol) with tris(tetra-*n*-butylammonium) hydrogen pyrophosphate according to the synthesis of GPP, as a white solid (213 mg, 55%). HRMS calc. 387.1852; found 387.1856. ^1^H NMR (400 MHz, D_2_O) δ ppm 3.91-3.99 (m, 2H), 1.49-1.67 (m, 3H), 0.94-1.38 (m, 14H), 0.84-0.94 (m, 12H). ^31^P NMR (162 MHz, D_2_O) δ ppm -10.24 (d, 19.40 Hz, P1), -10.82 (d, 19.40 Hz, P2).

**(2*E*,6*E*)-3,7,11-trimethyl-2,6,10-dodecatrien-1-yl methylenediphosphonate (FPP-Mu3)**

FPP-Mu3 was prepared from intermediate **8** (222 mg, 1.0 mmol) and tris (tetra-n-butylammonium) hydrogen methanediphosphonat according to the synthesis of GPP, as a white solid (152 mg, 40%). HRMS calc. 379.1573; found 379.1577. ^1^H NMR (400 MHz, D_2_O) δ ppm 5.41 (t, *J* = 7.2 Hz, 1H), 5.17 (q, *J_1_* = 9.01 Hz, *J_2_* = 8.20 Hz, 2H), 4.40 (t, *J* = 6.90 Hz, 2H), 2.24-1.86 (m, 10H), 1.68 (s, 3H), 1.66 (s, 3H), 1.59 (s, 6H). ^31^P NMR (162 MHz, D_2_O) δ ppm 17.88 (s, P1), 15.66 (s, P2).

**(2*E*,6*E*)-3,7,11-trimethyl-2,6,10-dodecatrien-1-yl hydroxyethylenediphosphona- the (FPP-Mu4)**

FPP-Mu4 was prepared from **8** (222 mg, 1.0 mmol) and tris (tetra-*n*-butylammonium) hydrogen ethylenediphosphonate according to the synthesis of GPP as a white solid (152 mg, 40% yield). as a white solid (144 mg, 35%). HRMS calc. 409.2481; found 461.2485. ^1^H NMR (400 MHz, D_2_O) δ ppm 5.46 (t, *J* = 6.60 Hz, 1H), 5.19 (m, 2H), 4.47 (dd, *J_1_* = *J_2_* = 6.64 Hz, 2H), 2.16-2.01 (m, 8H), 1.71 (s, 3H), 1.68 (s, 3H), 1.62 (s, 6H), 1.21 (s, 3H). ^31^P NMR (162 MHz, D_2_O) δ ppm 19.85 (d, *J* = 38.88 Hz, P1), 18.46 (d, *J* = 38.88 Hz, P2).

**Synthesis of BPH-652, BPH-754 and GGT2-I**

The synthesis of these compounds are illustrated in **Scheme 3-5** according to published methods [3-5].

**Scheme 3*^a^***

*^a^* Reagents and conditions: (a) triethyl phosphonoacetate, NaH, THF, 0^o^C; (b) Pd/C (5%), H_2_, RT, overnight; (c) LiAlH_4,_ THF, 0^o^C, 1 h; (d) MsCl, Et_3_N, NaI, 60^o^C, 1 h; (e) cyclohexyl diethylphosphonomethylsulfonate, NaH, THF, 0^o^C; (f) NH_3_, MeOH; (g) TMSBr, CH_3_CN, RT, 24 h, then MeOH, 30 min.

**Scheme 4*^a^***

*^a^* Reagents and conditions: (a) 1-bromodecane, K_2_CO_3_, CH_3_CN, 70°C, overnight; (b) 3N NaOH, RT, 1 h; (c) H_3_PO_3_, PCl_3_, tetramethylene sulfone, 75°C, 4 h; then H_2_O, 100°C, 2 h.

**Scheme 5*^a^***

*^a^* Reagents and conditions: (a) D-tyrosine methyl ester hydrochloride, pyridine, DMAP, reflux, 3 d; (b) BH_3_, THF, reflux, overnight; (c) 4-methoxybenzenesulfonyl chloride, pyridine, 0°C to RT, 18 h; (d) 1-methylimidazole-5-carboxaldehyde, trifluoroacetic acid, trifluoroacetic anhydride, triethylsilane, RT, 16 h; (e) 5-formyl-2-furylboronic acid, Pd(PPh_3_)_4_, K_2_CO_3_, DME/H_2_O = 2/1.

**1-(Diethoxyphosphoryl)-4-(3-phenoxyphenyl) butane-1-sulfonic acid (BPH-652)** BPH-652 was synthesized according to a published method. In brief, there are eight steps for the preparation of BPH-652 from the corresponding 3-phenoxybenzaldehyde **(15)**, which was reacted with triethylphosphonoacetate to obtain an unsaturated carboxylate. Then 1-(3-iodopropyl)-3-phenoxybenzene **(16)** was made from unsaturated carboxylate that was hydrogenated, reduced and further reated with NaI. Compound **16** was reacted with the sodium cyclohexyl diethylphosphonomethylsulfonate to give the triester **17**. Finally, **17** was deprotected and hydrolyzed to give the compound BPH-652 in 45% yield. HRMS calc. 387.0611; found 387.0615. ^1^H NMR (400 MHz, CDCl_3_) δ ppm 6.70-7.30 (m, 9H), 2.91-3.01 (m, 1H), 2.53-2.62 (m, 2H), 1.91-1.98 (m, 2H), 1.60-1.64 (m, 2H). ^31^P NMR (162 MHz, D_2_O) δ ppm 14.3.

**Hydroxy-2-(3-decyloxyphenyl)ethylidene-1,1-bisphosphonic acid (BPH-754)**

A solution of methyl 2-(3-hydroxyphenyl) acetate (**18**, 332 mg, 2.0 mmol), 1-bromodecane (396 mg, 2.4 mmol) and K_2_CO_3_ (552 mg, 4.0 mmol) in CH_3_CN was stirred overnight at 70°C. Then the mixture was filtered and concentrated in vacuum to give the residue **19**, which could be used for the next step without further purification. Compound **20** was made from **19** by being hydrolyzed with 3 N NaOH (1 mL) in methanol (5 mL) at room temperature for 1 h. Then acidification with 3 N HCl, methanol was removed, and the resulting residue filtered and washed with water. Finally, H_3_PO_3_ (250 mg, 3.0 mmol) and PCl_3_ (280 mg, 2.0 mmol) were added to a solution of **9b** (292 mg, 1.0 mmol) in 2 mL tetra methylene sulfone on stirring. The contents of the flask were stirred at 75°C for 4 h. After cooling to room temperature, water (5 mL) was added and the mixture was stirred further at 100°C for 2 hours. Then the solvent removed by evaporation and the residue was washed repeatedly by ether, acetonitrile, acetone and ethanol until the white solid was afforded in 45% yield. HRMS calc. 439.1652; found 439.1656. ^1^H NMR (400 MHz, MeOD), δ (ppm) 7.15 (t, *J* = 8.02 Hz, 1H), 6.88-6.90 (m, 2H), 6.72 (dd, *J*_1_ = 8.01 Hz, *J*_2_ = 2.45 Hz, 1H), 3.95 (t, *J* = 6.44 Hz, 2H), 3.19 (td, *J*_1_ = 16.80 Hz, *J*_2_ = 6.06 Hz, 2H), 1.72-1.79 (m, 2H), 1.42-1.49 (m, 2H), 1.33-1.36 (m, 12H), 0.90 (t, *J* = 8.04 Hz, 3H). ^31^P NMR (162 MHz, MeOD), δ (ppm) 21.57.

1. **[(3*R*)-3-[(4-hydroxyphenyl)methyl]-4-[(4-methoxybenzene)sulfonyl]-1-[(1-methyl-1H-imidazol-5-yl)methyl]-2,3,4,5-tetrahydro-1H-1,4-benzodiazepin-7-yl]furan-2-carbaldehy-de (GGT2-I)**

7-bromo-1,4-benzodiazepine-2,5-dione (**22**) was prepared with condensation reaction from 5-bromoisatoic anhydride (**21**) with D-tyrosinemethyl ester. The compound **22** was reduced to **23** by borane in THF for 16 h. Compound **24** was obtained via selective N-sulfonylation to introduce the sulfonamide moiety. Subsequently, the imidazolylmethyl group was introduced by reductive amination to afford **25**. Then GGT2-I can be prepared with a Pd(PPh3)4 mediated Suzuki couplings of **25** and 5-formyl-2-furylboronic acid, in a yield of 55%. HRMS calc. 613.2113; found 613.2101. ^1^H NMR (400 MHz, MeOD) δ (ppm) 9.23 (s, 1H), 7.32 (s, 1H), 7.30 (d, *J* = 2.22 Hz, 1H), 7.23 (dd, *J_1_* = 8.61 Hz, *J_2_* = 2.24 Hz, 1H), 7.21 (d, *J* = 3.82 Hz, 1H), 7.17 (d, *J* = 8.90 Hz, 2H), 6.71 (d, *J* = 8.28 Hz, 2H), 6.60 (d, *J* = 3.72 Hz, 1H), 6.51 (d, *J* = 8.90 Hz, 2H), 6.49 (d, *J* = 8.36 Hz, 2H), 6.44 (s, 1H), 6.26 (d, *J* = 8.42 Hz, 1H), 4.42 (d, *J* = 17.64 Hz, 1H), 4.35 (d, *J* = 17.64 Hz, 1H), 4.01 - 3.98 (m, 1H), 3.89 (d, *J* = 16.38 Hz, 1H), 3.56 (d, *J* = 16.56 Hz, 1H), 3.54 (s, 3H), 3.40 - 3.34 (m, 1H), 3.33 (s, 3H), 2.78 (dd, *J_1_* = 15.32 Hz, *J_2_* = 4.14 Hz, 1H), 2.60 (dd, *J_1_* = 13.76 Hz, *J_2_* = 4.98 Hz, 1H), 2.36 (dd, *J_1_* = 13.52 Hz, *J_2_* = 8.16 Hz, 1H). ^13^C NMR (100 MHz, MeOD) δ (ppm) 168.0, 153.7, 151.2, 146.6, 142.3, 141.2, 129.5 , 122.5, 121.0, 120.1, 119.1, 118.8, 118.1, 117.5, 117.4, 116.3, 115.9, 111.2, 106.5, 106.2, 104.4, 97.2, 59.6, 55.0, 52.2, 45.8, 44.8, 36.6, 30.8.

**References:**

1. Davisson VJ, Woodside AB, Neal TR, Stremler KE, Muehlbacher M, Poulter CD. Phosphorylation of Isoprenoid Alcohols. J Org Chem. 1986;51(25):4768-79. doi: DOI 10.1021/jo00375a005. PubMed PMID: WOS:A1986F366700005.

2. Roe SJ, Oldfield MF, Geach N, Baxter A. A convergent stereocontrolled synthesis of [3-C-14]solanesol. J Labelled Compd Rad. 2013;56(9-10):485-91. doi: 10.1002/jlcr.3083. PubMed PMID: WOS:000325088900016.

3. Song YC, Lin FY, Yin FL, Hensler M, Poveda CAR, Mukkamala D, et al. Phosphonosulfonates Are Potent, Selective Inhibitors of Dehydrosqualene Synthase and Staphyloxanthin Biosynthesis in Staphylococcus aureus. J Med Chem. 2009;52(4):976-88. doi: 10.1021/jm801023u. PubMed PMID: WOS:000263530200011.

4. Chen CKM, Hudock MP, Zhang YH, Guo RT, Cao R, No JH, et al. Inhibition of geranylgeranyl diphosphate synthase by bisphosphonates: A crystallographic and computational investigation. J Med Chem. 2008;51(18):5594-607. doi: 10.1021/jm800325y. PubMed PMID: WOS:000259342700014.

5. Stigter EA, Guo Z, Bon RS, Wu YW, Choidas A, Wolf A, et al. Development of Selective, Potent RabGGTase Inhibitors. J Med Chem. 2012;55(19):8330-40. doi: 10.1021/jm300624s. PubMed PMID: WOS:000309643500012.
