# Supplementary figures and images for: Farnesyl pyrophosphate is a new danger signal inducing acute cell death
Source: PLoS Biol. 2021 Apr 26;19(4):e3001134. doi: 10.1371/journal.pbio.3001134 (PMC8075202; doi:10.1371/journal.pbio.3001134)

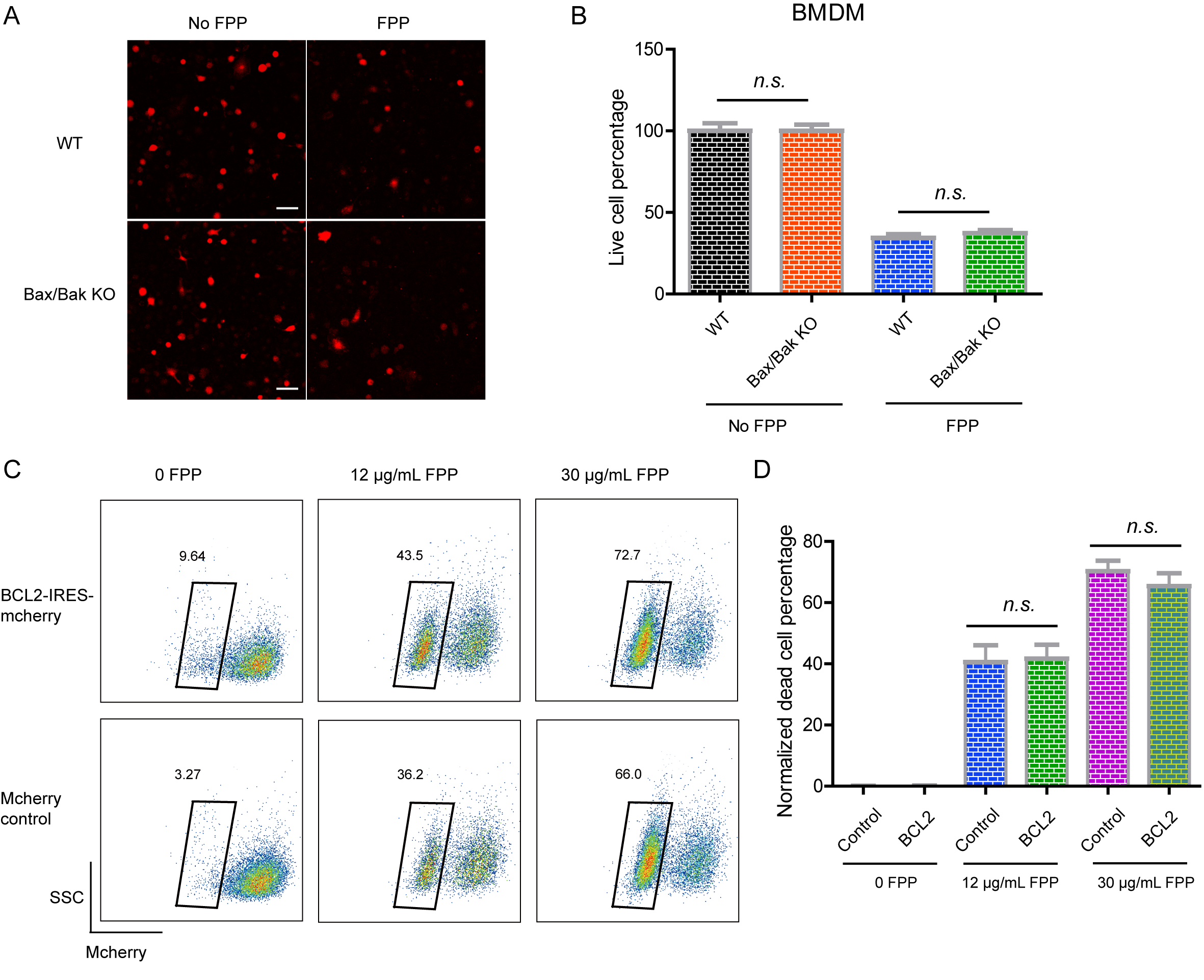

Supplement: S1 Fig — (A, B) Bax/Bak KO in BMDM did not affect FPP-induced cell death. Cre-IRES-mcherry were transfected into WT or Bax/Bak flox BMDM, then treated with 100 μg/mL FPP in the presence of Ca2+ for 1h and check the mcherry positive cell number change. (A) showed the representative images and (B) showed the statistical results. The scale bar is 100 μm. (C, D) Bcl2 overexpression did not affect FPP-induced cell death. Bcl2-IRES-mcherry and control mcherry was transfected into P815 cells. The mcherry positivity of Bcl2 group is 6% higher than that of control group. After being treated with FPP for 40 minutes, the mcherry fluorescence were checked with flow cytometry, and mcherry negative cells were counted as dead cells. Unpaired t test is used, and n.s. indicates p > 0.05. The original data in this figure except S1C Fig can be found in S2 Data. The original data in S1C Fig can be found in S5 Data. BMDM, bone marrow–derived macrophage; FPP, farnesyl pyrophosphate; KO, knockout; WT, wild-type. (TIF) [file pbio.3001134.s002.tif]

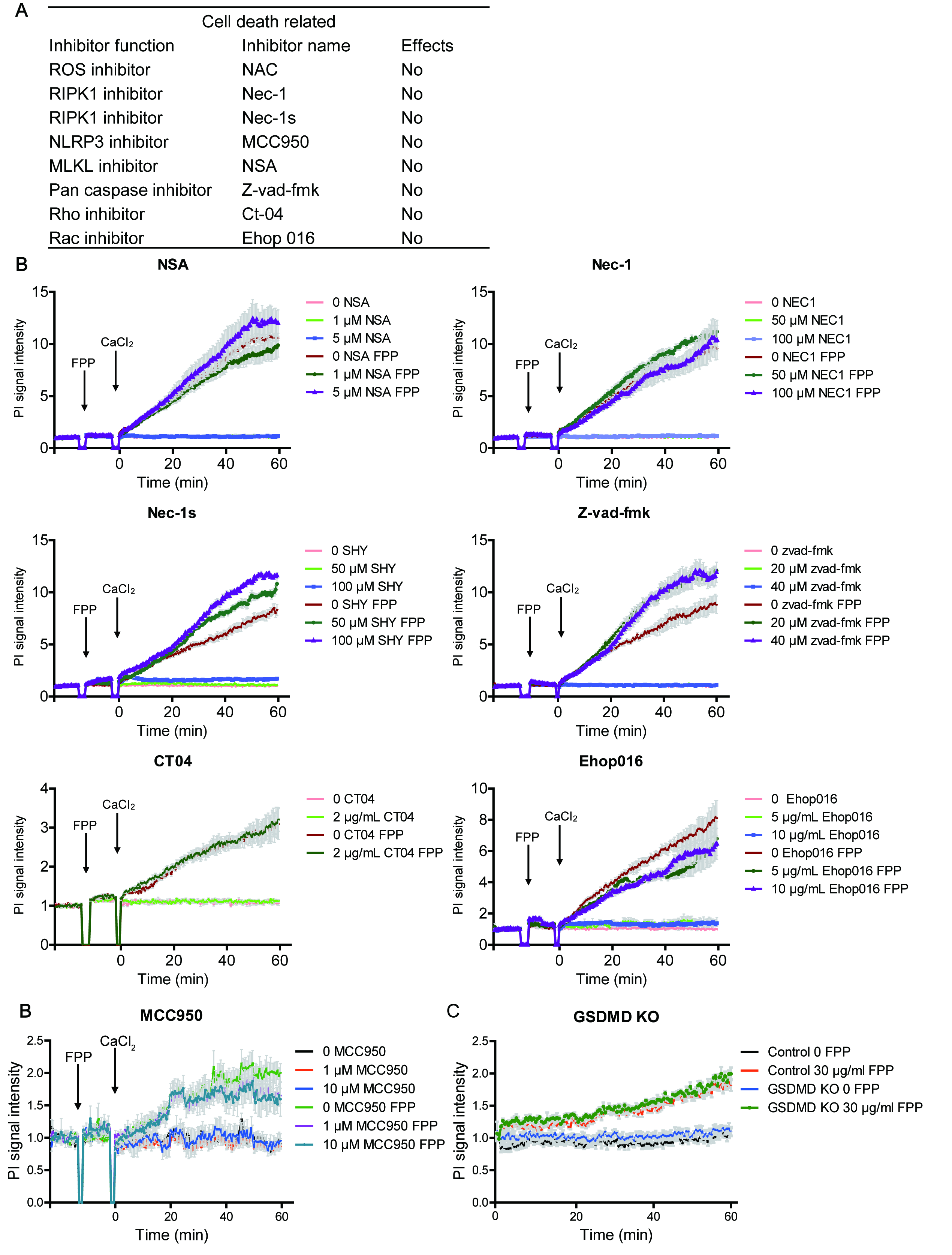

Supplement: S2 Fig — (A) Table with different inhibitors used, their functions, and their effects on FPP-induced cell death. (B) Effects of inhibitors targeting conventional cell death pathway, caspase, and GTPase on FPP-induced cell death in P815 cells. Significant difference between 50 μM Nec-1s FPP and 0 Nec1s FPP shows up from 27 minutes on (p < 0.05). Significant difference between 100 μM Nec-1s FPP and 0 Nec1s FPP shows up from 23 minutes on (p < 0.05). Significant difference between 20/40 μM Zvad-fmk FPP and 0 Zvad-fmk FPP shows up from 30 minutes on (p < 0.05). All the other inhibitors treatments show no significant differences compared to the corresponding controls (p > 0.05). Two-way ANOVA analysis is used. (C) GSDMD KO cannot significantly inhibit FPP-induced cell death. Spleen cells from WT and GSDMD KO mice were cultured in the presence of LPS. Moreover, 24 hours later, cells were treated with different concentrations of FPP, and the PI signal changes were measured. Each treatment had 3 to 4 replicates. Bars denote mean ± SEM (SEM in gray). Two-way ANOVA analysis is used, and no significant differences are observed between the experimental and corresponding control (p > 0.05). All the original data can be found in S2 Data. FPP, farnesyl pyrophosphate; KO, knockout; PI, propidium iodide; WT, wild-type. (TIF) [file pbio.3001134.s003.tif]

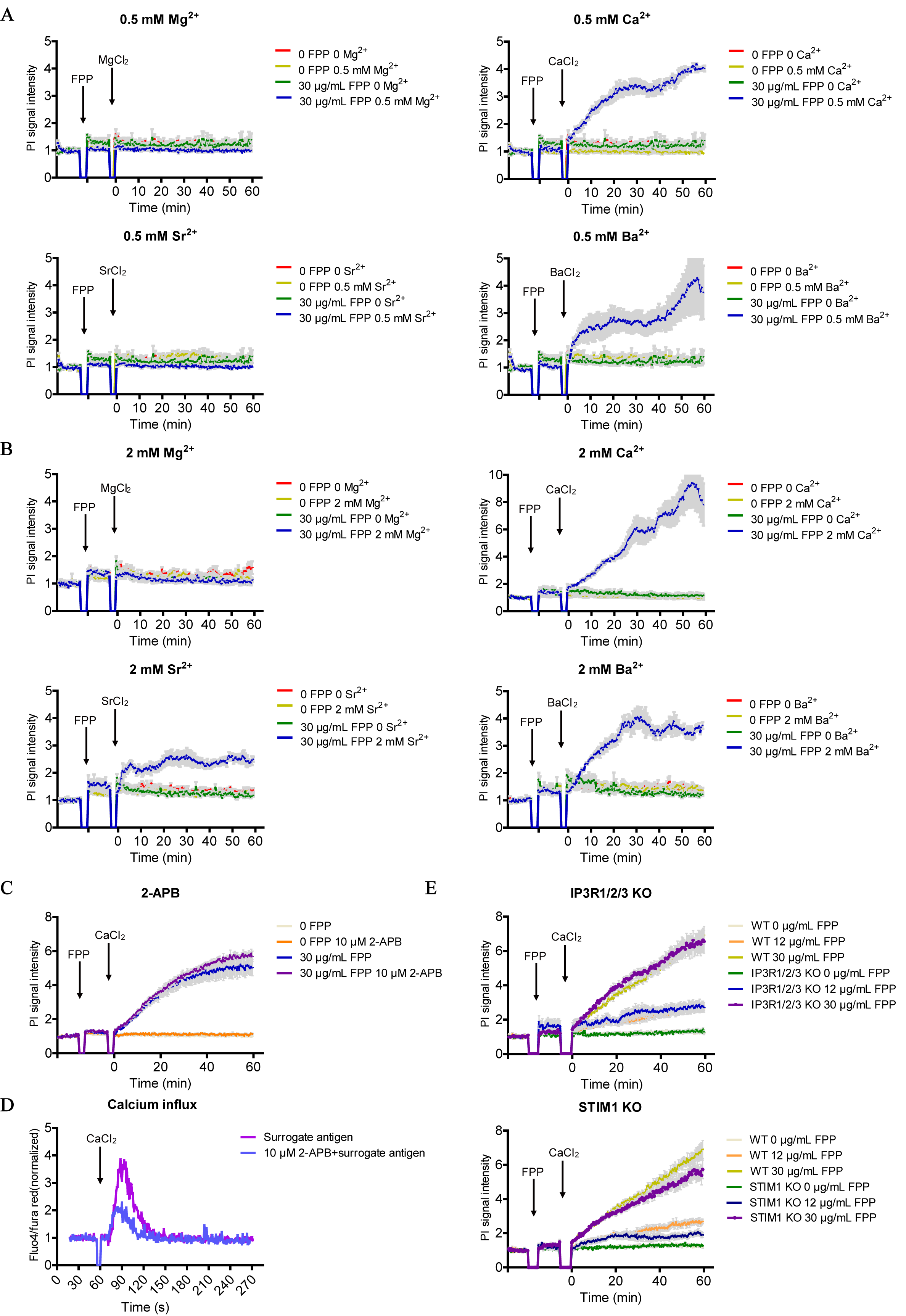

Supplement: S3 Fig — (A, B) FPP-induced cell death in the presence of different cations including Ca2+, Mg2+, Sr2+, and Ba2+ at 2 different concentrations, (A) 0.5 mM and (B) 2 mM. Significant difference between 30 μg/mL FPP 0.5 mM Ca2+ and the control shows up from 5 minutes on (p < 0.05). Significant difference between 30 μg/mL FPP 0.5 mM Ba2+ and the control shows up from 17 minutes on (p < 0.05). Significant difference between 30 μg/mL FPP 2 mM Ca2+ and the control shows up from 5 minutes on (p < 0.05). Significant difference between 30 μg/mL FPP 2 mM Ba2+ and the control shows up from 8 minutes on (p < 0.05). All the others show no significant difference (p > 0.05). (C) Effects of ER calcium depletion inhibitor 2-APB on FPP-induced cell death in P815 cells. No significant differences are observed between the 2-APB treated and untreated group (p > 0.05). (D) 2-APB effects on calcium influx after DT40 cells were stimulated with anti-IgG(H+L). (E) PI signal changes after FPP treatment in control cells and IP3R1/2/3 KO and STIM1 KO DT40 cells. Black arrows indicate the time points at which different reagents were added. Bars denote mean ± SEM (SEM in gray). No significant differences are observed between the KO and WT cells (p > 0.05). Two-way ANOVA analysis is used. All the original data can be found in S2 Data. ER, endoplasmic reticulum; FPP, farnesyl pyrophosphate; KO, knockout; PI, propidium iodide; WT, wild-type. (TIF) [file pbio.3001134.s004.tif]

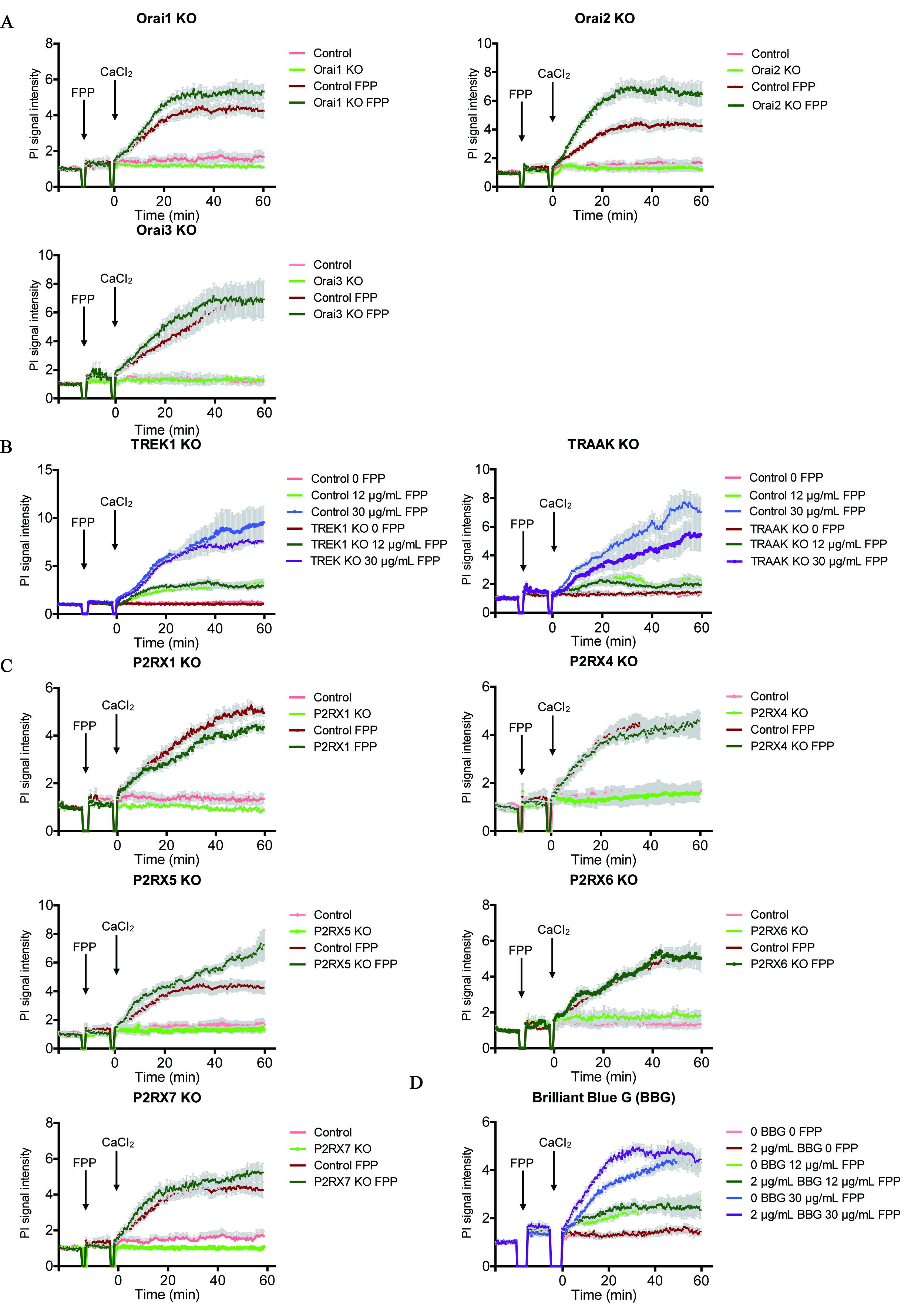

Supplement: S4 Fig — (A) PI signal changes after FPP treatment in control cells and ORAI1/2/3/ KO cells. Significant difference between Orai2 KO FPP and the control shows up from 15 minutes on (p < 0.05). All the others show no significant difference (p > 0.05). (B) PI signal changes after FPP treatment in control cells and TREK1/TRAAK KO cells. No significant differences are observed between the KO and WT cells (p > 0.05). (C) PI signal changes after FPP treatment in control cells and P2RX1/4/5/6/7 KO cells. Significant difference between P2RX5 KO FPP and the control shows up from 44 minutes on (p < 0.05). All the others show no significant difference (p > 0.05). (D) PI signal changes after FPP treatment in the presence of P2RX7 blocker brilliant blue G. Black arrows mean the time points at which different reagents are adding. Bars denote mean ± SEM (SEM in gray). No significant difference is observed between the inhibitor treated and untreated group (p > 0.05). Same controls used in Orai1 KO, Orai2 KO, P2RX5 KO, and P2RX7 KO because that are from 1 experiment. Data are representative of at least 2 independent experiments in (A) to (D). Two-way ANOVA analysis is used. All the original data can be found in S2 Data. FPP, farnesyl pyrophosphate; KO, knockout; PI, propidium iodide; WT, wild-type. (TIF) [file pbio.3001134.s005.tif]

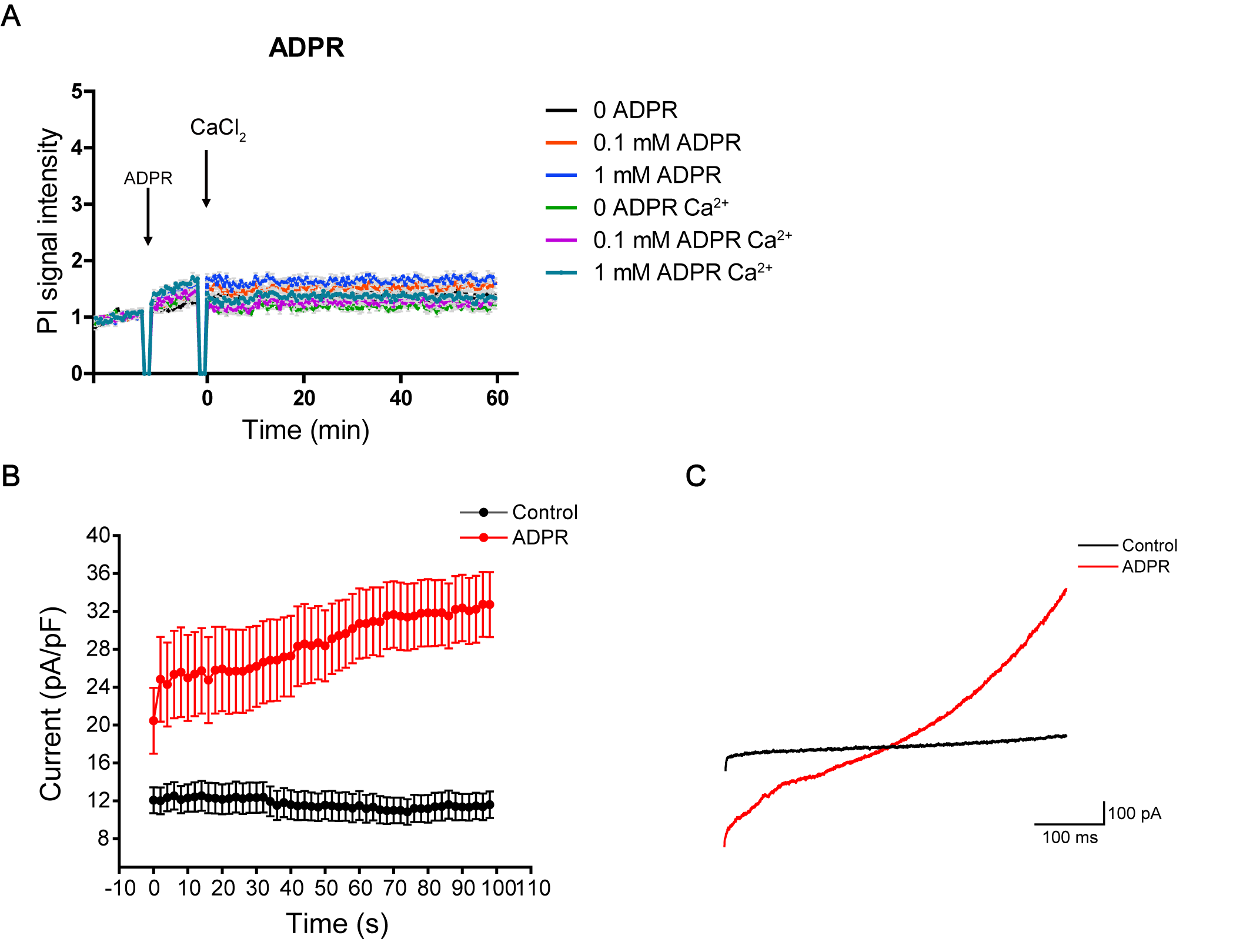

Supplement: S5 Fig — (A) ADPR cannot lead to acute cell death. P815 cells were resuspended in SLH buffer, and different concentrations of ADPR and 2 mM CaCl2 were added sequentially. PI signal intensity was measured during the process. Each treatment had 3 to 4 replicates. Bars denote mean ± SEM (SEM in gray). The first black arrow indicates the addition of ADPR, and the second black arrow indicates the addition of CaCl2. Two-way ANOVA analysis is used and no significant differences are observed between the ADPR treated and non-treated group (p > 0.05). (B) ADPR elicit TRPM2 current in TRPM2 overexpressed 293T cells. Current change of TRPM2 channels after intracellular addition of 200 μM ADPR (ADPR) or the control intracellular buffer without ADPR (Control). (N = 16 for Control; N = 16 for ADPR) This experiment is done according to the method published by Du et al. [34]. (C) The representative TRPM2 current trace elicited by the voltage ramp after ADPR stimulation. All the original data can be found in S2 Data. ADPR, adenosine diphosphate ribose; PI, propidium iodide; TRPM2, transient receptor potential melastatin 2. (TIF) [file pbio.3001134.s006.tif]

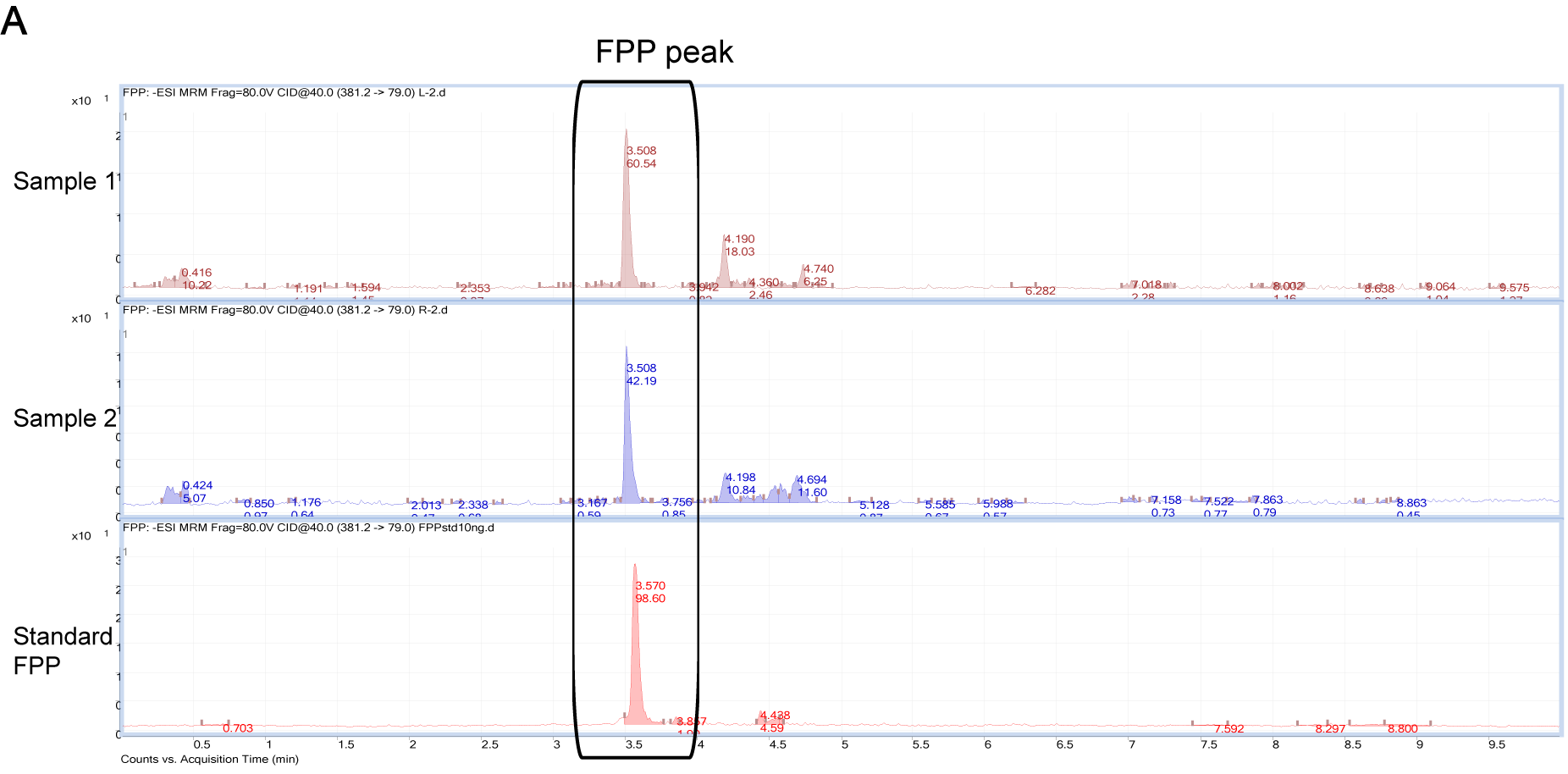

Supplement: S6 Fig — FPP, farnesyl pyrophosphate. (TIF) [file pbio.3001134.s007.tif]
