# Supplementary material for: Farnesyl pyrophosphate is a new danger signal inducing acute cell death
Source: PLoS Biol. 2021 Apr 26;19(4):e3001134. doi: 10.1371/journal.pbio.3001134 (PMC8075202; doi:10.1371/journal.pbio.3001134)

**Figure 1D**


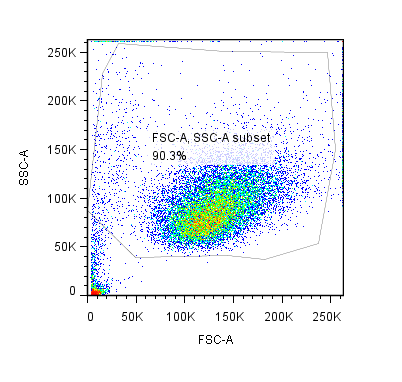
Step 1: gate out cells using FSC and SSC parameters


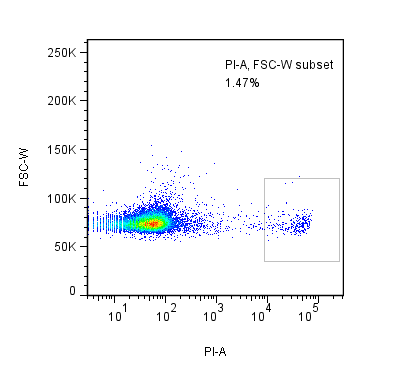
Step 2: gate out dead cells by gating PI positive cells

**Figure 3C**

Step 1: gate out cells using FSC and SSC parameters


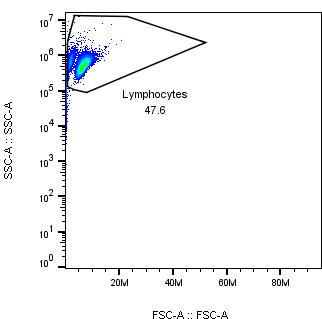


Step 2: change the graph to histogram and using FL2 (mitosox channel) as the X axle


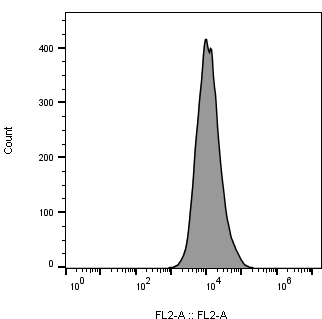


**Figure 3D**

Step 1: gate out cells using FSC and SSC parameters


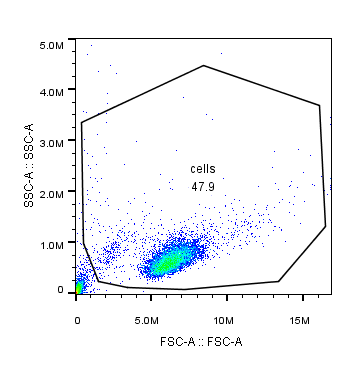
Step 2: gate out low red/green signal population to indicate the cell population which loses the mitochondrial potential.


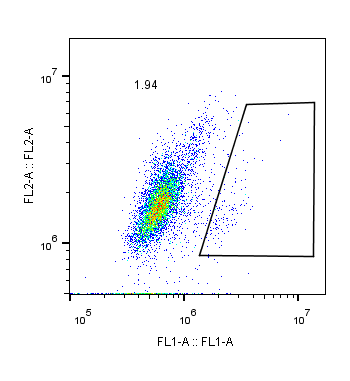


**Figure 4E**

Step 1: gate out cells using FSC and SSC parameters


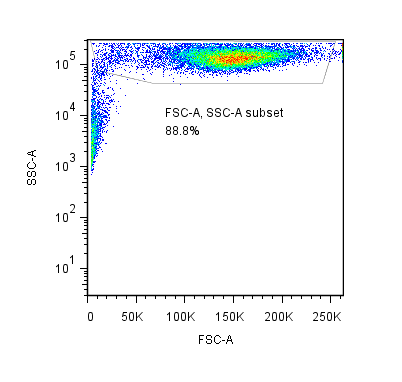


Step 2: gate out dead cells by gating PI positive cells


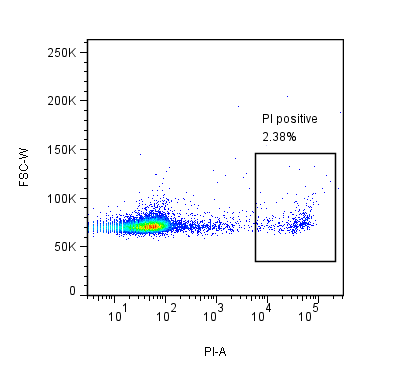


**Figure 4F**

Step 1: gate out cells using FSC and SSC parameters


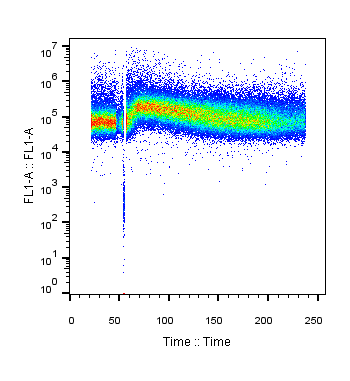
Step 2:
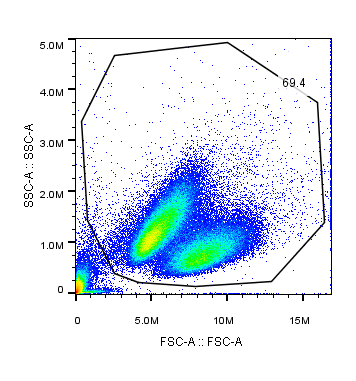
 change the X axle to “Time” and the Y axle to FL1 (Fluo4 channel)

**Figure S1C**

Step 1: gate out cells using FSC and SSC parameters


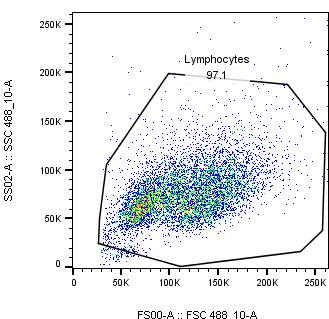


Step 2: gate out dead cells by gating mcherry negative cells


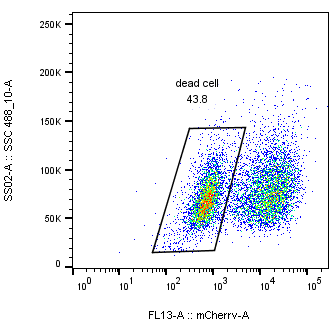

Supplement: S3 Data — (ZIP) [file pbio.3001134.s012.zip › gating strategy.docx]
